# Pregabalin mitigates microglial activation and neuronal injury by inhibiting HMGB1 signaling pathway in radiation-induced brain injury

**DOI:** 10.1186/s12974-022-02596-7

**Published:** 2022-09-21

**Authors:** Zhan Zhang, Jingru Jiang, Yong He, Jinhua Cai, Jiatian Xie, Minyi Wu, Mengdan Xing, Zhenzhen Zhang, Haocai Chang, Pei Yu, Siqi Chen, Yuhua Yang, Zhongshan Shi, Qiang Liu, Haohui Sun, Baixuan He, Junbo Zeng, Jialin Huang, Jiongxue Chen, Honghong Li, Yi Li, Wei-Jye Lin, Yamei Tang

**Affiliations:** 1 Department of Neurology, Sun Yat-Sen Memorial Hospital, Sun Yat-Sen University, Guangzhou, 510120 China; 2 Brain Research Center, Sun Yat-Sen Memorial Hospital, Sun Yat-Sen University, Guangzhou, 510120 China; 3 Guangdong Provincial Key Laboratory of Malignant Tumor Epigenetics and Gene Regulation, Medical Research Center, Guangdong-Hong Kong Joint Laboratory for RNA Medicine, Sun Yat-Sen Memorial Hospital, Sun Yat-Sen University, Guangzhou, 510120 China; 4 Radiotherapeutic Department, the Second Affiliated Hospital of Guangzhou Medical University, Guangzhou, China; 5 Key Laboratory of Brain, Cognition and Education Science, Ministry of Education, Institute for Brain Research and Rehabilitation, South China Normal University, Guangzhou, 510631 China; 6 MOE Key Laboratory of Laser Life Science & Institute of Laser Life Science, College of Biophotonics, South China Normal University, Guangzhou, 510631 China; 7 Guangdong Province Key Laboratory of Brain Function and Disease, Zhongshan School of Medicine, Sun Yat-Sen University, Guangzhou, China

**Keywords:** Radiation-induced brain injury, Microglia activation, Neuroinflammation, Neuron injury, HMGB1

## Abstract

**Background:**

Radiation-induced brain injury (RIBI) is the most serious complication of radiotherapy in patients with head and neck tumors, which seriously affects the quality of life. Currently, there is no effective treatment for patients with RIBI, and identifying new treatment that targets the pathological mechanisms of RIBI is urgently needed.

**Methods:**

Immunofluorescence staining, western blotting, quantitative real-time polymerase chain reaction (Q-PCR), co-culture of primary neurons and microglia, terminal deoxynucleotidyl transferase dUTP nick-end labeling (TUNEL) assay, enzyme-linked immunosorbent assay (ELISA), and CRISPR–Cas9-mediated gene editing techniques were employed to investigate the protective effects and underlying mechanisms of pregabalin that ameliorate microglial activation and neuronal injury in the RIBI mouse model.

**Results:**

Our findings showed that pregabalin effectively repressed microglial activation, thereby reducing neuronal damage in the RIBI mouse model. Pregabalin mitigated inflammatory responses by directly inhibiting cytoplasmic translocation of high-mobility group box 1 (HMGB1), a pivotal protein released by irradiated neurons which induced subsequent activation of microglia and inflammatory cytokine expression. Knocking out neuronal HMGB1 or microglial TLR2/TLR4/RAGE by CRISPR/Cas9 technique significantly inhibited radiation-induced NF-κB activation and pro-inflammatory transition of microglia.

**Conclusions:**

Our findings indicate the protective mechanism of pregabalin in mitigating microglial activation and neuronal injury in RIBI. It also provides a therapeutic strategy by targeting HMGB1-TLR2/TLR4/RAGE signaling pathway in the microglia for the treatment of RIBI.

**Supplementary Information:**

The online version contains supplementary material available at 10.1186/s12974-022-02596-7.

## Introduction

Radiotherapy is an important adjuvant therapy for patients with head and neck tumors, primary or metastatic brain tumors [1, 2]. However, radiotherapy also causes damage to the adjacent normal brain tissue and leads to central nervous system (CNS) complications [2, 3]. The clinical symptoms of radiation-induced brain injury (RIBI) include cognitive impairment and necrosis of brain tissues, which seriously affects the quality of life of patients [3, 4]. Due to lack of understanding to the pathogenesis of RIBI, limited options are available for clinical treatment of patients with RIBI [5]. Conventionally, corticosteroids and bevacizumab, an antibody targeting vascular endothelial growth factor (VEGF), have been proven to be effective in alleviating radiation-induced brain edema [6, 7]. However, due to the high recurrence rate and serious adverse reactions of these two treatments, it is urgently needed to develop new therapeutic intervention for RIBI [7, 8].

The main pathological characteristics of RIBI include excessive activation of glial cells, neuroinflammation, immune cell infiltration, blood–brain barrier (BBB) destruction, neuron loss, and memory and cognitive dysfunction [9, 10]. Microglia are the resident immune cells that play key role in immune surveillance and maintaining the homeostasis of the CNS [11, 12]. It has been suggested that microglia are very sensitive to radiation [13]. A few hours after radiation, microglia become rapidly activated and release pro-inflammatory cytokines, such as TNF-α, IL-1β, IL-6, and COX-2, which participate in tissue injury and neurogenesis impairment in the CNS [13, 14]. Therefore, inhibiting destructive inflammatory response induced by radiation may be of therapeutic potential for the treatment of RIBI.

Pregabalin, a structural derivative of gamma-aminobutyric acid (GABA), was developed as a potential first-line treatment for neuropathic pain [15]. It binds to the α2-δ subunit of synaptic voltage-gated calcium channels (VGCC) in the CNS [16]. By blocking VGCC, pregabalin significantly reduces the influx of Ca^2+^, so as to inhibit the release of excitatory neurotransmitters [17]. Increasing evidence has shown that pregabalin also plays a neuroprotective role in spinal cord injury, epilepsy, multiple sclerosis, traumatic brain injury, cerebral ischemia–reperfusion, diabetic retinopathy, fibromyalgia syndrome and other disease models [18–23]. Our previous work has demonstrated that pregabalin is effective and safe in the treatment of radiotherapy-induced neuropathic pain [24]. However, whether and how pregabalin may play a neuroprotective role in RIBI remains unclear.

Here, we reported that pregabalin treatment effectively inhibited neuroinflammation and prevented neuronal injury in the RIBI mouse model. Moreover, we demonstrated that pregabalin directly acted on neurons and inhibited cytoplasmic translocation of high-mobility group box 1 (HMGB1), a nuclear protein that is released from the cell in response to damage or stress [25]. Knocking down neuronal HMGB1 effectively alleviated irradiated neurons-induced microglia activation and inflammatory cytokine expression. Our findings also indicated the therapeutic potential of neutralizing HMGB1 or blocking its microglial receptors, including TLR2, TLR4, and RAGE, for the treatment of RIBI.

## Methods

### Animals

Adult male BALB/c mice at the age of 10–12 weeks used in this study were obtained from the Laboratory Animal Center of the Sun Yat-Sen University. All mice were housed in a climate-controlled room maintained on a 12-h light/dark cycle with food and drink ad libitum. After radiation and drug treatment, we observed the status of mice and recorded their body weight every day to monitor the health status of mice. All experimental procedures were approved by the Institutional Animal Care and Use Committee of the Sun Yat-sen University.

### Cranial irradiation and drug administration

The protocol for mouse irradiation has been previously described [13, 26]. Briefly, BALB/c male mice at 10–12 weeks of age were first separated randomly into four groups: for two non-irradiation groups, one was injected with saline solution (Con), and the other one was injected with pregabalin (PGB), and for the two irradiated groups, one was injected with saline solution (Rad), and the other one was injected with pregabalin (Rad + PGB). Both irradiated and non-irradiated control groups were deeply anesthetized, and cranial irradiation was conducted using the RS-2000 X-ray irradiator (Rad Source) as previously described with some modifications [13]. Anesthetized mice were fixed on a custom-designed platform. The head of each mouse was placed in an irradiation area (2 × 2 cm^2^) within the confines of the whole brain, from the post-canthus line to the post-aurem line, while other parts of the mouse body were shielded by lead plates and protected from exposure to radiation. A single dose of 30 Gy was delivered to mice at a rate of 3 Gy/min for 10 min, with a source-to-skin distance of 100 cm. Mice from the control group also underwent the same anesthesia procedures as those in the radiation group, but did not receive irradiation.

For pregabalin treatment, a dose of 30 mg/kg/day pregabalin was used in vivo study. First, Pregabalin (Pfizer Pharmaceuticals, New York, NY, USA) was diluted to final concentration of 1.5 mg/mL in normal saline (NS, 0.9% NaCl), which was prepared freshly at the time of injection. Pregabalin was injected intraperitoneally at 10:00 a.m. every day for 3, 7 and 14 consecutive days at a dose of 30 mg/kg body weight, starting from the day the mice were irradiated. The mice were injected at a total volume of 20 µl per gram body weight and the saline-only injection was used in the non-treated control groups. The body weight of mice was recorded daily. At each time point of irradiation and pregabalin treatment, a total of 30 mice received irradiation and 30 mice underwent a sham operation, whereas half of the mice in the irradiated and sham groups received pregabalin treatment. The left and right hemispheres were separated, half of which were separated into cortex and hippocampus, and total RNA was extracted by Trizol solution. The other hemisphere of the brains was placed in 4% paraformaldehyde solution for the subsequent immunofluorescence staining.

### Cell culture

For primary neuronal culture, the cortex and hippocampus were isolated from newborn Balb/c mice in ice-cold HBSS buffer (Gibco, 14,175,095). The brain tissues were cut into 1 cm^3^ pieces after removing the meninges. Then the tissue pieces were digested with 0.25% trypsin–EDTA (Gibco, 25,200–072) at 37 ℃ for 30 min and 10% fetal bovine serum was used to stop digestion. After filtration and centrifugation, cells were collected and cultured in neurobasal medium (Gibco, 21,103,049) supplemented with glutamax (Gibco, 35,050,061), B27 supplement (Gibco, 17,504,044), and pen strep (Gibco, 15,140,122) as previously described [27]. Primary microglia cultures derived from newborn Balb/c mice were prepared from mixed glial cultures using the “shaking off” method as previously described [13]. Primary microglia and the immortalized murine microglia BV2 cell line were cultured in DMEM/F12 medium (Gibco, C11330500BT) supplemented with penicillin and streptomycin (Gibco, 15,140–122) and 10% fetal bovine serum (Gibco, 10099141C).

According to previous report [13], a single dose of 10 Gy X-ray radiation effectively activated microglia and was used in this study [13]. Cells were replaced with the complete culture medium and treated with different concentrations of pregabalin (from 1 µM to 50 µM) immediately after irradiation. After 24 h of pregabalin treatment, the cells were used for immunofluorescence staining or real-time quantitative PCR analysis. The culture supernatant was used for ELISA detection.

### Co-culture of primary neurons and microglia

Primary neurons isolated from cortex and hippocampus of neonatal mice were cultured in 24-well plates. After the neurons grow to 10–14 days, microglia were added to the Transwell system (Corning, 3422) used for indirect co-culture model studying neuron and microglia crosstalk. Pregabalin (10 µM) was immediately added to the co-culture medium after radiation (10 Gy). 24 h later, the co-culture supernatant was collected for ELISA analysis to detect the relevant pro-inflammatory cytokines. Q-PCR analysis was used to study the mRNA expressions of cytokines in microglia and neurons. Immunofluorescence staining was used to study morphological changes of microglia and neurons after fixation and blocking.

### Quantitative real-time polymerase chain reaction (Q-PCR)

The tissues and cells were lysed with Trizol reagent (Invitrogen, 15,596,018). About 1 µg of total RNA was used for reverse transcription into cDNA by PrimeScript RT MasterMix (TaKaRa, RR036A) in a total volume of 20 µL. Q-PCR was performed on 50–100 ng cDNA of each sample in Multiplate™ 96-well PCR Plates (BIO-RAD, MLL9601) on a CFX96 Touch Real-Time PCR Detection System, and the final reaction volume was 20 µl. The data were analyzed by CFX Maestro Software (BIO-RAD). We used Q-PCR to detect and quantify the mRNA levels of *Tnf-α*, *Inos*, *Il-1β*, *Cox-2*, *Il-6*, *Icam-1*, *Hmgb1*, *Cgrp*, *Tac1*, *Mmp2*, *Mmp9*, *Cx3cl1*, *Ccl2*, and *Ccl21*. *β-actin* was used as a housekeeping gene to normalize the mRNA levels of target genes among different groups. All primers used in this work are shown in Additional file 1: Table S1.

### Immunofluorescence staining

At the time point of detection, the half brain was directly fixed in 4% paraformaldehyde (Biosharp, BL539A) overnight after perfusing with saline solution. After dehydration with gradient sucrose solution (from 20 to 30%), the brain samples were cut into 30 µm thick coronal sections with freezing microtome (Leica, CryoStar NX50). Then the slices were permeabilized with 0.1% Triton X-100 solution and blocked with 5% BSA (Sigma, A1933), followed by incubation with primary antibodies overnight at 4 ℃. After washing three times with PBS, the slices were incubated with specific Alexa Fluor-coupled secondary antibodies for 1 h at room temperature. Finally, the slices were incubated with DAPI to label the nucleus. The primary antibodies include rat anti-CD68 (BIO-RAD, MCA1957), rabbit anti-IBA1 (Wako, 019–19,741), rabbit anti-IL-6 (CST, 12912S), rabbit anti-TNF-α (CST, 11948S), mouse anti-NeuN (Merck Millipore, MAB377), rabbit anti-NeuN (Merck Millipore, ABN78), rabbit anti-MAP2 (Proteintech, 17,490–1-AP), rabbit anti-Caspase-3 (CST, 9662S), rabbit anti-HMGB1 (CST, 3935S), and mouse anti-Beta Tubulin (Proteintech, 66,240–1-Ig). For the detailed information of all primary antibodies used in the study, including the manufacturers, species of origin, catalog number, application and dilution ratio, see Additional file 1: Table S2. The immunofluorescence-stained brain slices were imaged by fluorescence microscope (Leica DM6B, Germany) and laser scanning confocal microscope (Nikon C2, Japan), for detailed image acquisition and analysis.

### Western blotting

Western blotting was conducted as described previously with some modifications [27]. Briefly, cells were lysed with an appropriate amount of RIPA lysis buffer (Beyotime, P0013B) on ice and centrifuged at 13,000 rpm for 15 min at 4 °C. The supernatants were collected and processed with 5 × SDS-PAGE loading buffer. The protein concentration of all samples was measured using the BCA assay (Thermo Scientific, 23,227). Protein samples (about 30 μg) were separated on 10% SDS-gel electrophoresis and transferred to PVDF membranes (Merck Millipore, ISEQ00010). Then, the membranes were incubated with the primary antibody overnight at 4 °C, followed by Alexa Fluor-conjugated secondary antibodies. Detection was performed using the Odyssey Infrared Imaging System (LI-COR, Biosciences, Lincoln, NE, USA).

### Terminal deoxynucleotidyl transferase dUTP nick-end labeling (TUNEL) assay

Apoptotic cells were detected by DeadEnd Fluorometric TUNEL System (Promega, G3250) according to the manufacturer’s instructions, as previously described [4]. Briefly, the slices were permeated in 20 g/ml protease K solution for 20 min and blocked in 5% BSA for 1 h. After washing 3 times with PBS, slices were soaked in equilibrium buffer for 10 min, and then incubated with TDT reaction mixture in the dark at 37 ℃ for 1 h. Finally, DAPI was stained to label the nucleus.

### Enzyme-linked immunosorbent assay (ELISA)

The levels of TNF-α and IL-1β in the cell culture supernatant were detected by ELISA according to the instructions of the manufacturer (Neobioscience, China). Before measuring the concentrations of TNF-α and IL-1β, we detected the total protein concentration of the supernatant using the BCA assay, which aimed to calculate the ratios of TNF-α or IL-1β to total proteins in the cell culture supernatant.

### gRNA design and generation

For gRNA design, NGG sites in the exon of the target gene were selected, cloned and sequenced. First, gRNA was designed with the CCTop tool [28]. In order to check the specificity of gRNA and potential off-target sites, gRNA sequences were searched using BLAST algorithm on the CCTop (https://cctop.cos.uni-heidelberg.de/) to ensure that all gRNAs are without any potential off-target sites in genome.

### Recombinant LentiCRISPRv2-Cas9-gene generation

LentiCRISPRv2 plasmid cleaved by restriction endonuclease was used for the connection of the target fragments. Oligonucleotide primers were designed according to the sequence of gRNA and the sequence of the enzyme digestion products. Detailed gRNA sequences are shown in Additional file 1: Table S3 and detailed oligonucleotide sequences are shown in Additional file 1: Table S4. After annealing, the oligonucleotides are connected with the incomplete vector by T4 DNA ligase (TAKARA, 2011A). After transformation and cloning, the correctness of the target gene plasmid was verified by sequencing.

### CRISPR–Cas9-mediated gene knockout

We knocked out HMGB1, TLR2, TLR4, and RAGE genes using CRISPR–Cas9 technique. Briefly, their gRNAs were recombined with lentiCRISPRv2 vector, respectively. The recombinant plasmid was sequenced and contrasted for monoclonal amplification. The empty lentivirus vector was used as a negative control. The plasmid was transferred into HEK-293 T cells to prepare lentivirus particles. The cell culture medium containing lentivirus was added to cells. After infection 24 h, the cells were replaced with the new culture medium and treated with puromycin 1 μg/ml for 3–5 days. Finally, the knockout efficiency of target gene in cells was confirmed by western blotting, immunofluorescence, and Q-PCR, which was used for subsequent study. Detailed primers used for the verification of CRISPR/Cas9-mediated gene knockout are shown in Additional file 1: Table S5.

### Statistical analysis

All data are presented as mean ± SEM. Statistical analyses were performed using GraphPad Prism (GraphPad Software, Inc.). All other groups were compared with the indicated group and at least three independent experiments were conducted in this study. Significant differences between groups were tested using the one-way ANOVA procedure, followed by the Student’s *t*-test using SPSS software (IBM) for 2-group comparisons. Differences were considered statistically significant at *p* < 0.05.

## Results

### Pregabalin inhibited microglia activation and inflammatory response in a mouse model of RIBI

In the animal model studies and clinical practice, both single irradiation with high dose and fractionated irradiation with low dose are reported and in practice [29, 30]. We selected a single dose of cranial irradiation for studying RIBI due to the advantages of stable pathological phenotypes such as glial activation, vascular damages, white matter changes and long-term cognitive impairment that can occur within few weeks post-irradiation [13, 31]. Compared with fractionated low-dose irradiation, a single high-dose irradiation also induced more pronounced and early onset of inflammatory response in the brain [32]. Our previous research has revealed that a single dose of 30 Gy radiation could trigger microglia activation and production of microglia-derived pro-inflammatory cytokines in the RIBI mice at the acute phase, which further led to neuronal injury [13, 33]. Whole brain irradiation with a dose of 30 Gy was performed in BALB/c mice in this study. To study the protective potential of pregabalin in the mouse model of RIBI, we considered the dose of pregabalin used in the clinical study as we have previously reported, that pregabalin at a dose of 150 mg per day was effective in reducing neuropathic pain [24]. We then calculated a dose of approximately 30 mg/kg for pregabalin treatment in mice based on an estimated body weight of 60 kg per capita in humans, an equivalent dose conversion between animals and humans based on the body surface area calculations, in which the dose administered to mice was about 12.3 times of that of humans [34]. Therefore, pregabalin was used at a dose of 30 mg/kg in this study.

**Fig. 1:**
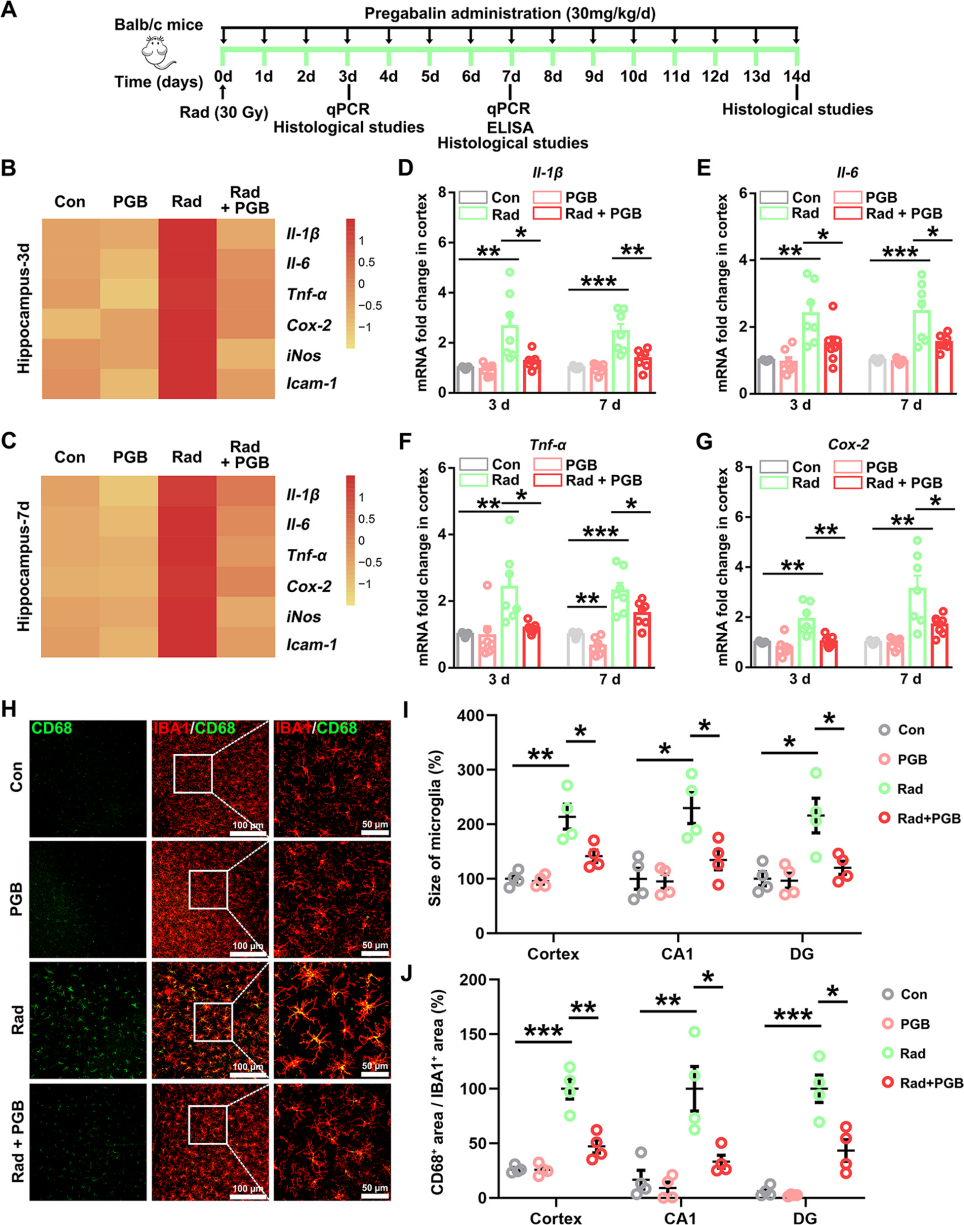
Pregabalin inhibited microglia activation and inflammatory response in the RIBI mice. **A** Timeline illustration of the experimental design. **B, C** The heat map showed the mRNA changes of the pro-inflammatory factors in the hippocampus of mice on day 3 and 7. **D**–**G** Q-PCR analysis of *Il-1β*, *Il-6*, *Tnf-α*, and *Cox-2* mRNA levels in the cortex of RIBI mice 3 and 7 days after pregabalin treatment. **H** Representative images of IBA1 and CD68 co-labeling in the cortex of RIBI mice 14 days after pregabalin treatment. **I**, **J** Microglial body size and the proportion of CD68^+^ area/IBA1^+^ area in the cortex and hippocampus (CA1 and DG) were quantified among the different groups. Data were analyzed by one-way ANOVA followed by the Student’s *t*-test analysis. All other groups were compared with the indicated group. n = 4–6 mice per group for the heat map analysis. n = 7 mice per group for Q-PCR analysis. *n* = 4 mice per group and 2–3 slices per mouse for immunofluorescence staining. Data were presented as mean ± SEM, *p < 0.05, **p < 0.01, and ***p < 0.001. Four groups were established for in vivo study: control mice (Con), control mice treated with pregabalin (PGB), RIBI-model mice (Rad), and RIBI-model mice treated with pregabalin (Rad + PGB)

Body weight of mice was recorded daily after treatment with pregabalin (30 mg/kg/day). Pregabalin showed a protective tendency against weight loss in irradiated mice (Additional file 1: Fig. S1). Mice were euthanized and analyzed on days 3, 7, and 14 after irradiation and pregabalin treatment, respectively (Fig. 1A). Since hippocampus is involved in memory formation and cognitive function [13], we isolated hippocampus to evaluate the changes of the pro-inflammatory factors. As shown in Fig. 1B, C, the mRNA levels of the pro-inflammatory factors, including *Il-1β, Il-6*, *Cox-2*, *Tnf-α*, *iNos*, and *Icam-1*, were significantly increased in the hippocampus 3 or 7 days after irradiation, consistent with previously published reports [13, 33]. Importantly, we observed that pregabalin treatment significantly reduced these pro-inflammatory cytokine expressions (Fig. 1B, C). Consistent effects of pregabalin on inhibiting the inflammatory factors expressions, including *Il-1β*, *Il-6, Tnf-α*, and *Cox-2*, were also found in the cortex of RIBI mice (Fig. 1D–G). These findings indicated that pregabalin could effectively inhibit the cerebral inflammatory response in the RIBI mice.

Increased pro-inflammatory cytokines after radiation were mainly attributed to microglia [13], so we next evaluated the pathological changes of microglia in the RIBI mice. As expected, the soma size of microglia in the cortex began to increase on the 3rd day after radiation, and became even larger in the following 7th and 14th days (Additional file 1: Fig. S2A, B). Similarly, the expression of CD68, a marker of activated microglia [35], was also gradually increased with the time after irradiation (Additional file 1: Fig. S2C), indicating that microglia changed from resting state to activated state. Moreover, pregabalin could significantly inhibit microglia activation 3 or 7 days after treatment, which is closely related to the changes of the pro-inflammatory cytokines (Additional file 1: Fig. S3A–D). Increased microglial body size and CD68 expression in both cortex and hippocampus (CA1 and DG regions) was also inhibited after 14 days of continuous treatment with pregabalin in the RIBI mice (Fig. 1H–J, Additional file 1: Fig. S4A, B). Together, these results indicated that pregabalin showed significant pharmacological effectiveness in repressing microglia activation and inflammatory response, implying that pregabalin might have the potential value in alleviating RIBI.

### Pregabalin protected neuron from radiative microglia-mediated damage

**Fig. 2:**
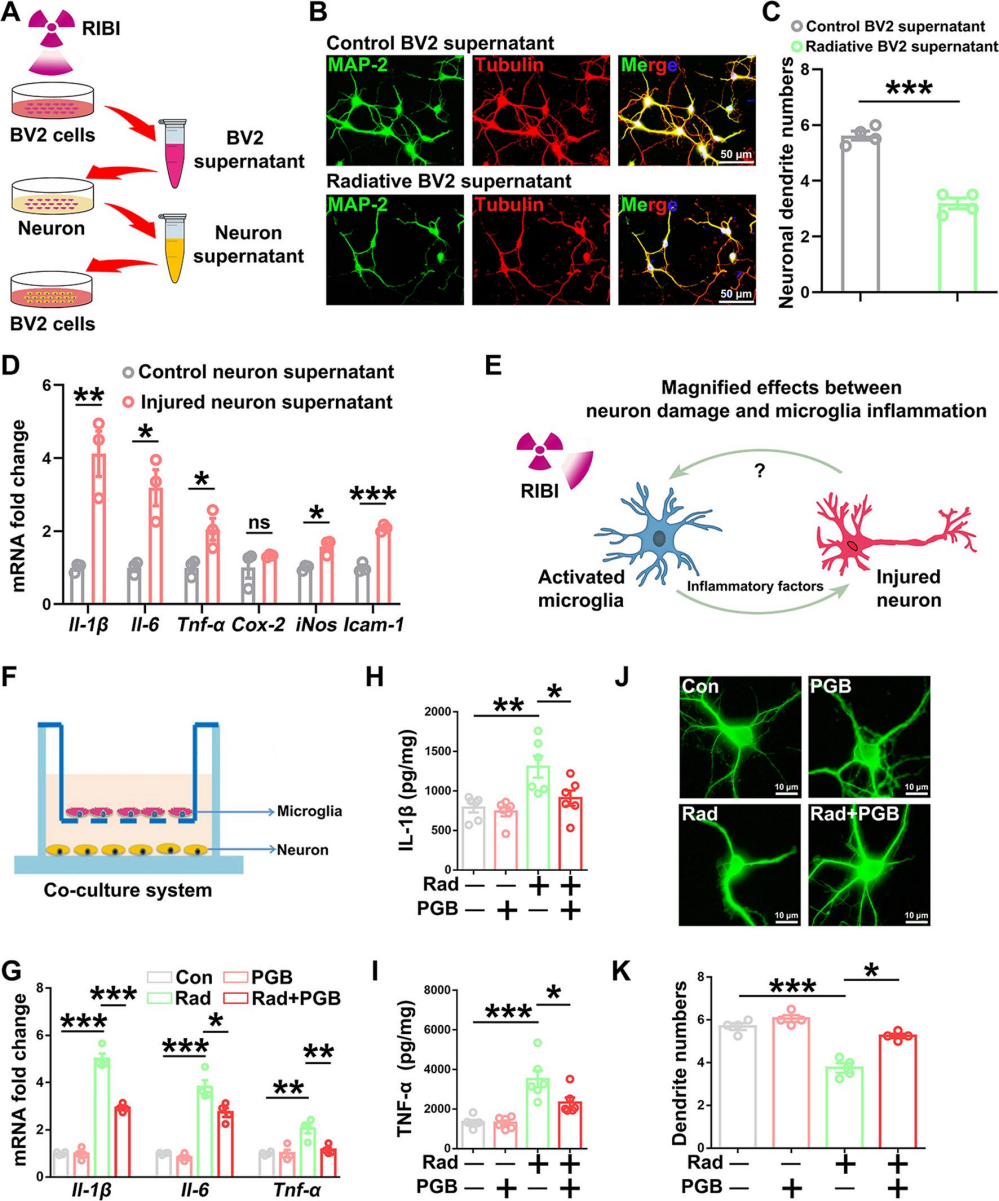
Pregabalin protected neuron from radiative microglia-mediated damage. **A** Schematic diagram of the in vitro experiments. **B** Representative images of MAP-2 and β-tubulin co-labeling in neurons. **C** Statistics of the neuronal dendric numbers among the different groups. **D** Q-PCR analysis of *Il-1β*, *Il-6*, *Tnf-α*, *Cox-2*, *iNos*, and *Icam-1* mRNA levels in BV2 cells incubated with supernatant from injured or non-injured neuron. **E** Schematic diagram of radiation-induced secondary damage of neurons. **F** Schematic diagram of the co-culture system between microglia and neurons. **G** Q-PCR analysis of *Il-1β*, *Il-6*, and *Tnf-α* mRNA levels in microglia after co-culture with neurons. **H-I** ELISA analysis of IL-1β and TNF-α protein levels in microglia after co-culture with neurons. **J** Representative images of MAP-2 and β-tubulin co-labeling in neurons after co-culture with microglia. **K** Statistics of the neuronal dendric numbers. Data were analyzed by one-way ANOVA followed by the Student’s *t*-test analysis. All other groups were compared with the indicated group. n = 3–6 for each group. Data were presented as mean ± SEM, ns = not significant, *p < 0.05, **p < 0.01, and ***p < 0.001. Four groups were established for in vitro study: control cells (Con), control cells treated with pregabalin (PGB), irradiated cells (Rad), and irradiated cells treated with pregabalin (Rad + PGB)

Increasing evidences suggest that microglia activation-mediated inflammatory response contributes to neuronal injury and neurodegenerative diseases [36, 37]. We next examine the effect of pregabalin in inhibiting the pro-inflammatory cytokines secretion from microglia. First, the effect of activated microglia induced by radiation on neuronal injury was evaluated in vitro (Fig. 2A). Neurons incubated with the culture supernatant of irradiated BV2 cells showed decreased number of neuronal dendrites in immunofluorescence staining of MAP2 (Fig. 2B, C), indicating that inflammatory cytokines produced by irradiated microglia could lead to neuronal damage. In order to test the possible crosstalk between injured neurons and microglia, non-irradiated BV2 cells were incubated with the culture supernatant from injured neurons that was previously incubated with supernatant of irradiated BV2 cells (Fig. 2A). Interestingly, we found that the inflammatory response of BV2 cells can be triggered after incubation with the culture supernatant from injured neurons, revealed by the elevation of mRNA levels of *Il-1β*, *Il-6*, *Tnf-α*, *iNos*, and *Icam-1* as compared with the non-injured neuron culture supernatant (Fig. 2D). Therefore, a feed-forward and amplifying effect was observed between inflammatory microglia and injured neurons after radiation (Fig. 2E). Importantly, pregabalin treatment in the co-culture system of microglia and neurons significantly blocked radiation-induced expression of pro-inflammatory cytokines, including *Il-1β*, *Il-6*, and *Tnf-α* mRNA levels in the BV2 cells and also secreted protein levels of IL-1β and TNF-α (Fig. 2F–I). Pregabalin also prevented radiation-induced reduction of neuronal dendrites (Fig. 2J–K). Together, our findings confirmed the protective effects of pregabalin in preventing radiation-induced inflammatory response of microglia and neuronal injury in the in vitro model of RIBI.

### Pregabalin inhibited inflammatory response by directly acting on neuron, rather than microglia or astrocyte

**Fig. 3:**
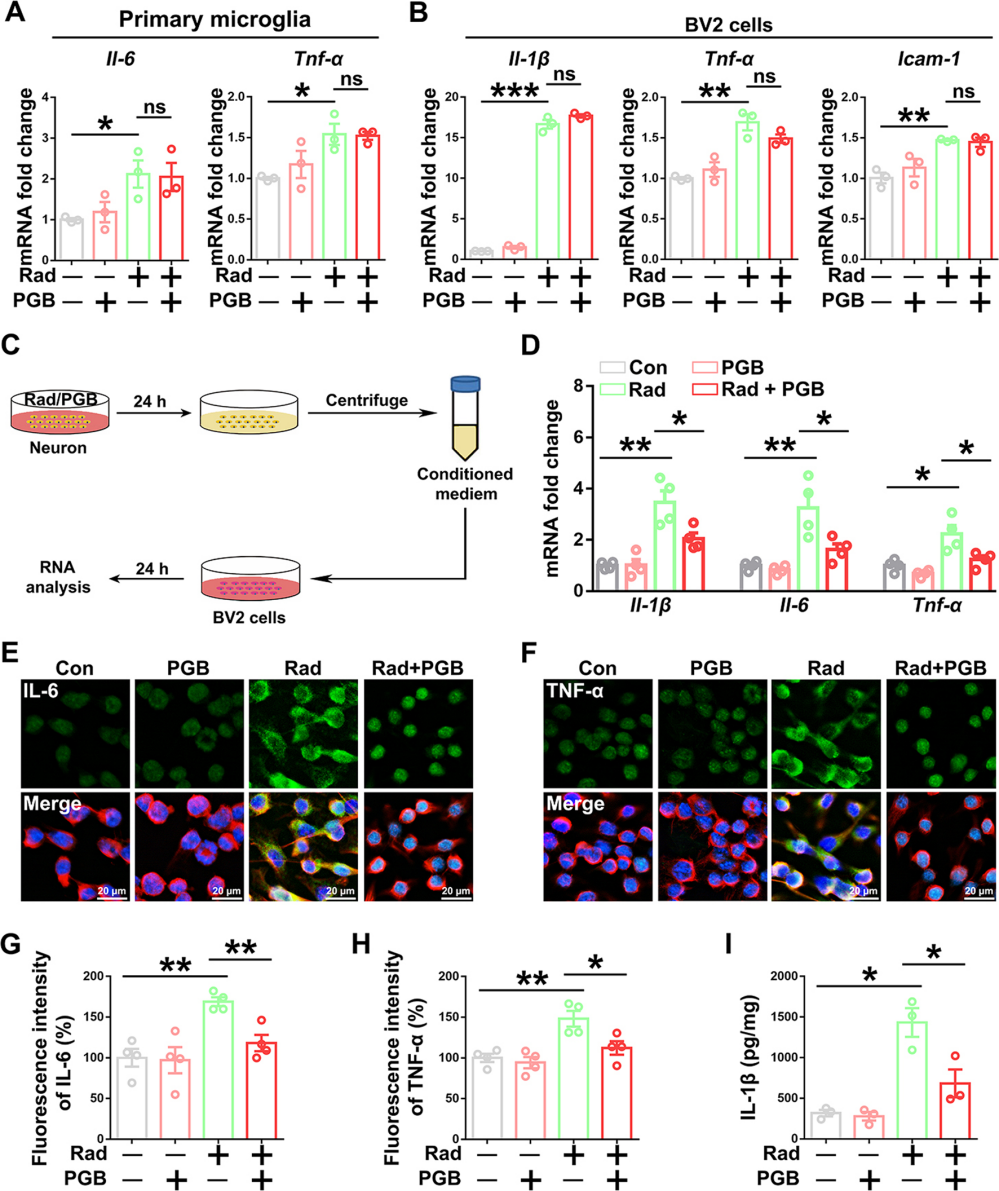
Pregabalin inhibited inflammatory response by directly acting on neurons, rather than microglia or astrocyte in vitro. **A** Q-PCR analysis of *Il-6* and *Tnf-α* mRNA levels in microglia with pregabalin treatment after radiation. **B** Q-PCR analysis the effects of pregabalin on *Il-1β*, *Tnf-α*, and *Icam-1* mRNA levels in BV2 cells after radiation. **C** Schematic diagram of microglia incubated with neuron supernatant. **D** Q-PCR analysis of *Il-1β*, *Il-6*, and *Tnf-α* mRNA levels in BV2 cells after incubated with the supernatant from pregabalin-treated neurons. **E–F** Representative immunofluorescent images of IL-6 (**E**) and TNF-α (**F**) in BV2 cells among the different groups. Staining with β-tubulin was used to visualize cytoskeleton. **G-H** The fluorescence intensity data of IL-6 (**G**) and TNF-α (**H**) were imaged by confocal microscopy and analyzed. **I** ELISA analysis of IL-1β protein level in the BV2 cells culture supernatant. Data were analyzed by one-way ANOVA followed by the Student’s *t*-test analysis. All other groups were compared with the indicated group. n = 3–4 for each group. Data were presented as mean ± SEM, ns = not significant, *p < 0.05, **p < 0.01, and ***p < 0.001

Since pregabalin effectively inhibited radiation-induced microglia activation and inflammatory response, we next focused on exploring its pharmacological mechanism, which should provide mechanistic insights for RIBI. First, primary microglia and immortalized BV2 cells from a murine microglial cell line were used for in vitro study. The mRNA levels of pro-inflammatory factors, such as *Il-1β*, *Il-6*, *Tnf-α*, or *Icam-1*, were significantly increased after a single dose 10 Gy irradiation, consistent with our previous findings in vivo (Fig. 3A, B). Several studies have shown that pregabalin could effectively inhibit NF-κB activation and cytokines secretion in the splenocytes, neuroblastoma, or glioma cells in vitro at a concentration of 1–25 µM [38, 39]. Pregabalin at a dose of 10 µM was firstly used to treat microglia in the study. Unexpectedly, increased mRNA levels of the pro-inflammatory cytokines were not affected after pregabalin treatment at different concentrations (1 µM, 6.25 µM, 12.5 µM, 25 µM, and 50 µM) in both irradiated BV2 cells and primary microglia (Fig. 3A, B, Additional file 1: Fig. S5A–D). Immunofluorescence staining further confirmed that pregabalin had no effect on radiation-induced increases in pro-inflammatory cytokines TNF-α and IL-6 protein levels in BV2 cells (Additional file 1: Fig. S6A–D). Thus, these results suggested that pregabalin was not directly acting on microglia to attenuate the inflammatory responses.

Since astrocytes and neurons, two important cell types in the CNS, are involved in the regulation of inflammatory responses of microglia [40, 41], we next asked whether neurons or astrocytes may be involved in regulating microglia activation after pregabalin treatment in RIBI. Supernatants of astrocytes or primary neurons were collected at 24 h after irradiation with or without pregabalin treatment, and were used to incubate BV2 cells for additional 24 h, followed by RNA extraction of BV2 cells (Fig. 3C, Additional file 1: Fig. S7A). Compared with the supernatant of non-irradiated neurons, irradiation-only neuronal supernatant could significantly upregulate the mRNA levels of pro-inflammatory factors including *Il-1β*, *Il-6*, and *Tnf-α* in BV2 cells (Fig. 3D). Of note, absence in the mRNA elevation of *Il-1β*, *Tnf-α*, and *Il-6* were observed in BV2 cells treated with supernatant from irradiated neurons with pregabalin treatment (Fig. 3D), rather than irradiated astrocyte with pregabalin treatment (Additional file 1: Fig. S7B–E). Upregulated *Il-1β* mRNA level was also observed in BV2 cells after treatment with the irradiation-only astrocyte supernatant (Additional file 1: Fig. S7B). Examination with immunofluorescence staining further confirmed the absence in IL-6 and TNF-α protein elevation in BV2 microglia after treatment with supernatant of pregabalin-treated neurons (Fig. 3E–H). Moreover, ELISA assay further showed that secreted IL-1β protein level from cultured BV2 cells were effectively inhibited after incubation with supernatant of pregabalin-treated neurons (Fig. 3I). Together, these results suggested that pregabalin acted on neurons to inhibit radiation-induced microglia activation and inflammatory responses in the RIBI mouse model.

### Pregabalin rescued neuronal apoptosis and loss in the RIBI mice

**Fig. 4:**
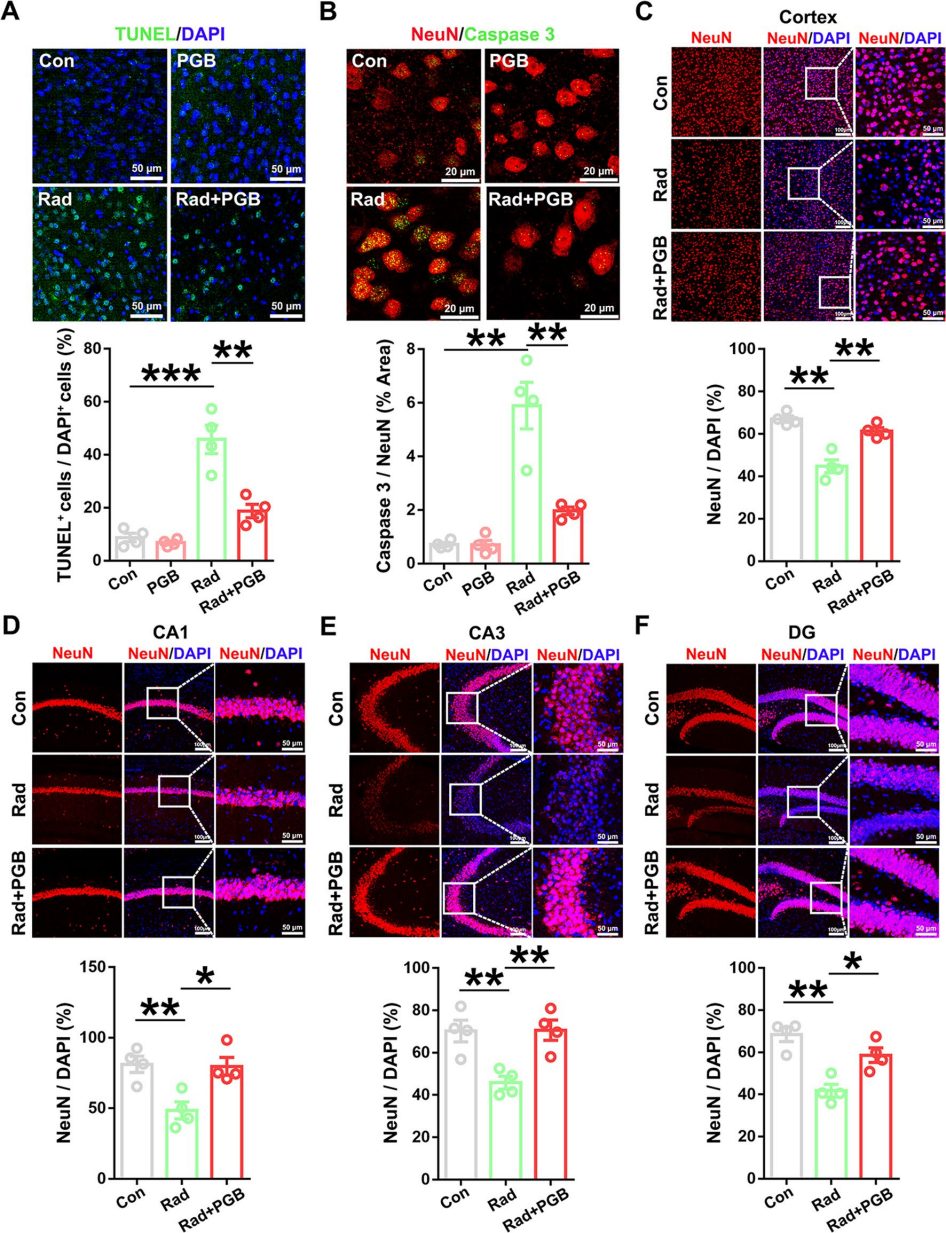
Pregabalin rescued neuronal apoptosis and loss in the RIBI mice. **A** Representative images of TUNEL staining and quantification of TUNEL-positive cells in the cortex. **B** Representative images and quantification of Caspase 3/NeuN immunofluorescence staining in the cortex. **C-F** Representative immunofluorescent images and quantification of neuronal proportion in the cortex and hippocampus. Data were analyzed by one-way ANOVA followed by the Student’s *t*-test analysis. All other groups were compared with the indicated group. n = 4 mice per group and 2–3 slices per mouse for immunofluorescence staining. Data were presented as mean ± SEM, *p < 0.05, **p < 0.01, and ***p < 0.001

To examine the protective effect of pregabalin in radiation-induced neuroinflammation and brain injury in vivo, TUNEL staining was used to evaluate neuronal injury after pregabalin treatment in the RIBI mice. Compared with the non-irradiated group, our data showed that TUNEL-positive apoptotic cells were increased in mice that received single exposure of 30 Gy irradiation (Fig. 4A). Notably, pregabalin treatment significantly reduced TUNEL-positive cells in the irradiated mice (Fig. 4A). Similar observation was observed as pregabalin significantly reduced caspase 3-labeled apoptotic neurons (NeuN-positive) after irradiation (Fig. 4B). Consistently, we also found that pregabalin restored neuronal number as calculated by NeuN^+^/DAPI^+^ cells after radiation in both cortex and hippocampus of irradiated mice (Fig. 4C–F). In conclusion, these results indicated that pregabalin effectively protected neurons from radiation-induced cell death.

### Pregabalin inhibited neuronal HMGB1 translocation from nucleus to cytoplasm in vivo and in vitro

**Fig. 5:**
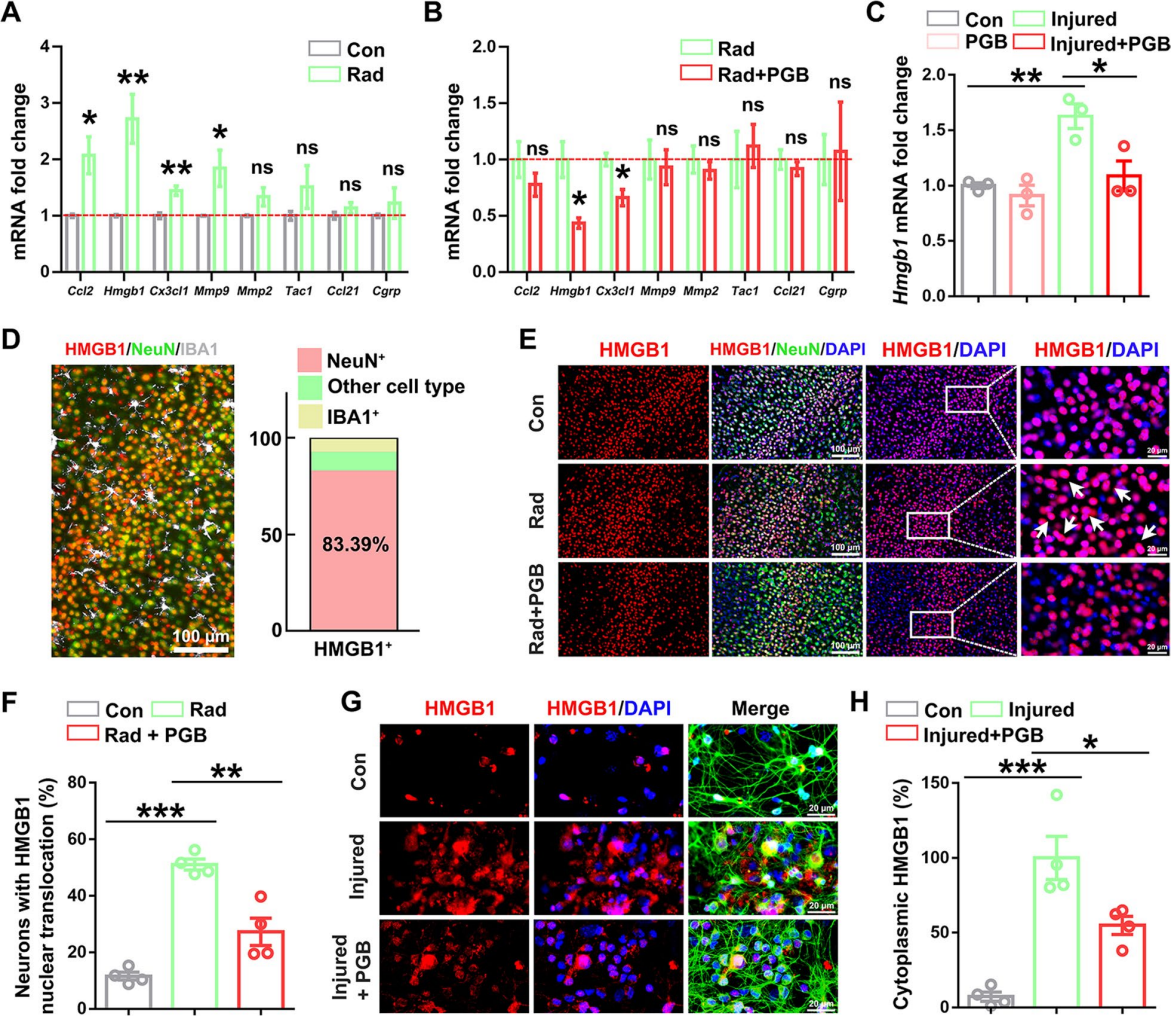
Pregabalin inhibited neuronal HMGB1 translocation from nucleus to cytoplasm in vivo and in vitro. **A-B** Q-PCR analysis of *Ccl2*, *Hmgb1*, *Cx3cl1*, *Mmp9*, *Mmp2*, *Tac1*, *Ccl21*, and *Cgrp* mRNA levels in the cortex. n = 4 mice per group for Q-PCR analysis in vivo. **C** Q-PCR analysis of *Hmgb1* mRNA level in neurons among the different groups. n = 3 per group for Q-PCR analysis in vitro. **D** Immunofluorescence staining and statistics showed that HMGB1 was mainly expressed in neurons. **E**, **F** Immunofluorescence staining and statistics of HMGB1 cytoplasmic translocation ratio in neurons in vivo. n = 4 mice per group and 2–3 slices per mouse for immunofluorescence staining. **G-H** Immunofluorescence staining and statistics of HMGB1 cytoplasmic translocation ratio in neurons in vitro. Staining with β‑tubulin (green) to visualize cytoskeleton. n = 4 mice per group for immunofluorescence staining in neurons. Data were analyzed by one-way ANOVA followed by the Student’s *t*-test analysis. All other groups were compared with the indicated group. Data were presented as mean ± SEM, ns = not significant, *p < 0.05, **p < 0.01, and ***p < 0.001

To study how pregabalin acts on neurons and participates in the inhibition of microglial inflammatory response, we firstly investigated the molecular profile of irradiated neurons whose expression can be altered by pregabalin treatment. Studies have shown that cytokines released from neurons, including CCL2, CCL21, HMGB1, CGRP, Substance P, CX3CL1, MMP2, and MMP9, can mediate microglia activation in brain injury, neuropathic pain, or neurodegenerative diseases [41, 42]. Among these disease-associated genes, Q-PCR analysis showed that *Ccl2*, *Hmgb1*, *Mmp9*, and *Cx3cl1* mRNA levels were obviously increased after irradiation, compared with the control group (Fig. 5A). Remarkably, the *Hmgb1* mRNA level was up-regulated by 2.72-fold after radiation, making it the most significantly changed factors among cytokines mentioned above. Importantly, pregabalin treatment effectively reduced *Hmgb1* mRNA level but not *Mmp2*, *Mmp9*, *Cgrp*, *Ccl2*, *Ccl21*, or *Tac1* gene expression in the irradiated mouse brains or in primary neurons treated with culture supernatant of irradiated BV2 cells (Fig. 5B, C, Additional file 1: Fig. S8A–G). In terms of *Cx3cl1*, the mRNA level was decreased in irradiated mice but showed none significance in primary neurons, indicating that pregabalin may target the expression of neuronal HMGB1 and repress radiation-induced microglia activation.

HMGB1 protein is mainly located in the nuclei of various cells, whose translocation to cytoplasm and secretion has been reported to participate in the signaling pathway of neuroinflammation [43]. Immunofluorescence staining showed that for all HMGB1-positive cells detected in the mouse cortex, the proportion of NeuN^+^ neurons was about 83.39%, while the proportion of other cells was only about 16.61% in which microglia was about 6.65% (Fig. 5D), indicating that HMGB1 was mainly expressed in the neurons. Notably, radiation induced HMGB1 translocation from nucleus to cytoplasm of cortical neurons, which can be reversed by pregabalin treatment (Fig. 5E, F). Consistent with the in vivo findings, cytoplasmic translocation of HMGB1 protein also occurred in the primary neurons after incubation with supernatant from irradiated BV2 cells (Fig. 5G), and pregabalin treatment effectively inhibited this neuronal HMGB1 translocation (Fig. 5G, H). Together, these results suggested that pregabalin inhibited HMGB1 cytoplasmic translocation of injured neurons and neuronal HMGB1 might be an important target for pregabalin-mediated mitigation of microglial inflammatory response in RIBI.

### Pregabalin mitigated NF-κB-mediated microglial inflammatory response by inhibiting neuronal HMGB1 cytoplasmic translocation and secretion

**Fig. 6:**
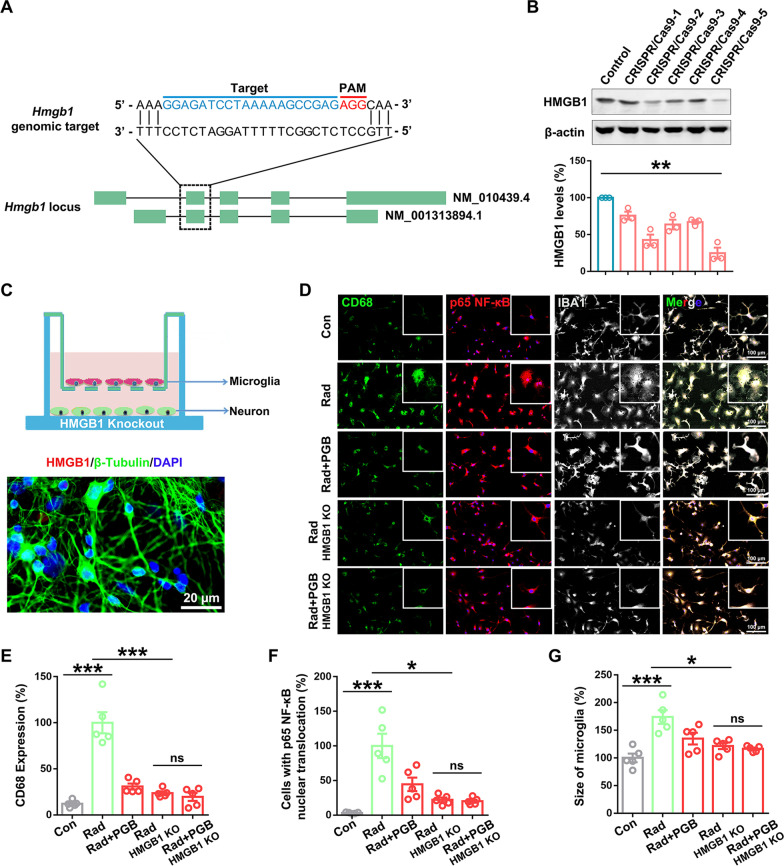
Pregabalin mitigated NF-κB-mediated microglial inflammatory response by inhibiting neuronal HMGB1 cytoplasmic translocation and secretion. **A** Schematic diagram of CRISPR/Cas9-mediated HMGB1 knockout in neurons. **B** Western blotting was used to detect the knockout efficiency among different HMGB1 gRNAs in neurons. n = 3 per group for Western blotting analysis. **C** Co-culture of microglia and neurons after HMGB1 knockout. **D** Immunofluorescence staining was used to detect CD68 expression, p65 NF-κB nuclear translocation, and the cell body size of primary microglia in the co-culture system. **E–G** Statistical analysis of CD68 expression, p65 NF-κB nuclear translocation, and the cell body size of microglia among the different groups. n = 5 per group for immunofluorescence staining in vitro. Data were analyzed by one-way ANOVA followed by the Student’s *t*-test analysis. All other groups were compared with the indicated group. Data were presented as mean ± SEM, ns = not significant, *p < 0.05, **p < 0.01, and ***p < 0.001

To verify that pregabalin inhibited microglial inflammatory response through the HMGB1-mediated signaling pathway, neuronal *Hmgb1* gene was knocked out using the CRISPR–Cas9 genome editing technique (Fig. 6A). As shown in Fig. 6B, the *Hmgb1* gRNA-5, with the highest efficiency for knocking out HMGB1 gene in neurons (up to 75%), was used for the subsequent study. Knocking out neuronal HMGB1 expression effectively inhibited the microglial nuclear translocation of p65 NF-κB from cytoplasm to nucleus in a co-culture system of microglia and neurons in vitro (Fig. 6C–E). Meanwhile, decreased microglial CD68 expression and decrescent average body size of microglia were also observed after neuronal HMGB1 knockout (Fig. 6F, G), which was consistent with the protective effects of pregabalin in vivo. Moreover, treatment with pregabalin after HMGB1 knockout did not further inhibit microglia activation (Fig. 6E–G), implying that HMGB1 is the downstream factor for the protective effect of pregabalin.

**Fig. 7:**
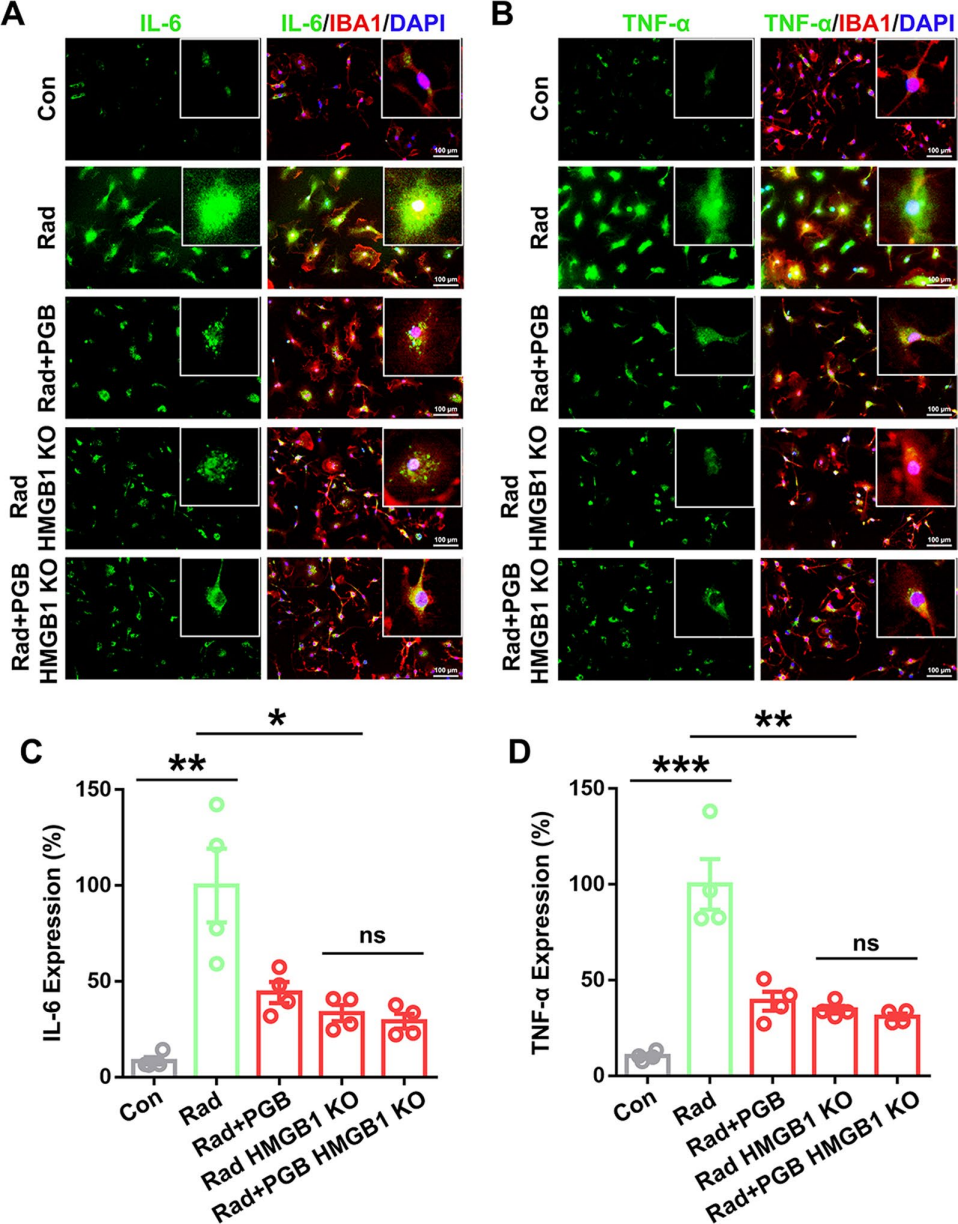
Pregabalin inhibited microglial inflammatory response through neuronal HMGB1 signaling pathway. **A**, **B** Immunofluorescence staining was used to detect microglial IL-6 and TNF-α expressions under different conditions in the co-culture system of neurons and microglia. **C**, **D** Statistical analysis of IL-6 and TNF-α expressions among the different groups. Data were analyzed by one-way ANOVA followed by the Student’s *t*-test analysis. All other groups were compared with the indicated group. n = 4 per group for immunofluorescence staining in vitro. Data were presented as mean ± SEM, ns = not significant, *p < 0.05, **p < 0.01, and ***p < 0.001

Immunofluorescence staining showed that microglial IL-6 and TNF-α expression was significantly increased after radiation in a co-culture system of neurons and microglia (Fig. 7A–D). Knocking out neuronal HMGB1 or pregabalin treatment significantly inhibited both TNF-α and IL-6 expression, which is consistent with above results alleviating microglial inflammatory response. Moreover, no significant differences were observed between pregabalin and vehicle-treated groups after HMGB1 knocking out (Fig. 7A–D), suggesting that pregabalin inhibited radiation-induced neuroinflammation and microglia activation by suppressing the mRNA expression, protein cytoplasmic translocation and secretion of neuronal HMGB1.

### TLR2, TLR4, and RAGE are responsible for HMGB1-mediated inflammatory signaling pathway in microglia

Previous studies implicated that HMGB1 can act on TLR2, TLR4, and RAGE receptors to induce pro-inflammatory cytokines expression and release from microglia [44–46]. Thus, RAGE, TLR2, and TLR4 genes were knocked out by CRISPR/Cas9-mediated gene editing in BV2 microglia cells (Additional file 1: Fig. S9A–C). The cells were subsequently treated with culture supernatant from radiation-injured or normal neurons for 24 h. We found that knocking out TLR2, TLR4, and RAGE significantly inhibited injured neuron-mediated increases in *Il-1β*, *Il-6*, and *Tnf-α* mRNA induction in BV2 cells (Additional file 1: Fig. S9D–F), indicating that HMGB1-TLR2/TLR4/RAGE signaling was mainly responsible for the inflammatory cascade of radiation-injured neuroinflammation. Together, these results suggested that pregabalin suppresses HMGB1-TLR2/TLR4/RAGE signaling pathway and interrupts pathological crosstalk between radiation-injured neurons and reactive microglia.

## Discussion

Radiotherapy is the first-line treatment for head and neck tumors [3]. However, it also carries the potential risks of radiation-induced brain injury, including focal brain necrosis, brain edema, epilepsy, cerebrovascular diseases, and cognitive dysfunction [47]. RIBI can be classified into acute, early delayed, and late delayed injury according to the time of disease onset, including morphological and functional defects [9]. Currently, available intervention and treatments for RIBI mostly focus on the late delayed symptoms. However, at this stage, the disease-associated symptoms, including gliosis, demyelination, vascular abnormalities, and white matter necrosis, are already irreversible [6]. There is still a lack of research targeting the pathological mechanisms at acute and early stages of RIBI. In this study, we revealed that pregabalin can effectively inhibit radiation-induced microglia activation and neuroinflammation at early stage of RIBI, which further alleviated neuronal injury and loss. Our findings therefore provide a preventive strategy and carry clinical relevance for the protection of patients after receiving radiotherapy for head and neck tumors.

Microglial activation and neuroinflammation are important pathological features of RIBI. As the innate immune cells in the brain, microglia are early responder after cranial irradiation, which releases pro-inflammatory factors, such as IL-6, IL-1β, and TNF-α, and causes damage to neuronal function, vascular integrity, neurogenesis, immunity homeostasis, and cognitive function [6, 13, 47]. Therefore, targeting radiation-induced neuroinflammation has been considered as a therapeutic strategy to treat RIBI [47]. Although traditional medicine like corticosteroids and bevacizumab can both alleviate the symptoms of RIBI to a certain extent, their effects on the improvement of neuroinflammation and microglia activation are not significant [4, 8]. Notably, our previous study demonstrated that pregabalin could alleviate radiotherapy-related neuropathic pain in the patients with RIBI [24]. Considering that microglia activation is involved in neuropathic pain, we therefore examined the protective effects of pregabalin on radiation-induced neuroinflammation. Here, through the combination of both in vivo and in vitro studies, we found that pregabalin can mitigate microglial inflammatory response by inhibiting radiation-induced HMGB1 cytoplasmic translocation and secretion from neurons, therefore preventing pro-inflammatory transition of microglia.

Microglia-mediated inflammation is considered to be a possible mechanism for the occurrence or deterioration of brain diseases including RIBI [41, 48]. However, how microglia are activated under pathological conditions remains largely under investigated. Traditional studies mainly focus on how external stimuli affect the morphology and molecules changes of microglia to trigger neuroinflammation. However, the interaction between microglia and other cells is also an integral part but often overlooked in the development of disease. It has been reported previously that the crosstalk between microglia and neuron is necessary for the sensing and maintenance of the physiological function of microglia and brain homeostasis [41]. Increasing evidences have shown that the pathological interaction between microglia and neurons can lead to synaptic dysfunction or neuronal loss, which contributes to the progression of certain brain diseases, but such case has not been reported in RIBI [49, 50]. Thus, investigating the crosstalk between neurons and microglia at acute phase of RIBI may provide mechanistic insight of the disease. In this study, we found that neuronal signaling changes could also drive microglia activation in RIBI. We provided a novel insight by targeting neuroinflammation in brain injury diseases.

To understand the abnormal changes in the communication between neurons and microglia, we used a mouse model of RIBI and identified HMGB1 was involved in the protective process of pregabalin against RIBI after screening a large number of cytokines released by neurons. In addition to the direct damage to neurons caused by radiation, radiation also resulted in neuronal HMGB1 cytoplasmic translocation and secretion, which activated microglia and resulted in the elevation of pro-inflammatory cytokine release and the subsequent neuronal damage. More importantly, our findings showed that pregabalin reduced microglia activation by inhibiting HMGB1-mediated microglial NF-κB inflammatory signaling pathway, and knocking out neuronal HMGB1 by CRISPR/Cas9 gene editing technique blocked the protective effect of pregabalin. As a common biomarker and potential target for traumatic brain injury (TBI), cerebral ischemia, stroke, epilepsy, and cognitive dysfunction, HMGB1 is involved in the neuroinflammation in these brain injury models [51]. In addition, blocking HMGB1 signaling pathway can protect early lung injury caused by radiation [52]. TLR2, TLR4, and RAGE, three receptors involved in HMGB1-induced inflammatory pathway in microglia, may promote strong inflammatory effects under the pathological condition [51]. Indeed, our results showed that by knocking out neuronal HMGB1, or microglial TLR2, TLR4, and RAGE, it also effectively inhibited the vicious cycle of radiation-induced microglia activation, neuroinflammation, and neuronal damage. Therefore, our results demonstrated the important mechanism of microglial activation under RIBI, which suggested that HMGB1-TLR2/TLR4/RAGE signaling pathway could be targeted for the prevention and treatment of RIBI.

Although ours and other studies have observed that HMGB1 is mainly expressed in neurons [25, 53], HMGB1 is also partially expressed in astrocytes and rarely in microglia [25, 54]. We therefore could not exclude the possible contribution of HMGB1 pathway from other cell types in the brains with RIBI, and whether pregabalin acts on other non-neuronal cell types to alleviate the pathological effects of HMGB1. In addition, TLR2 and TLR4 are important members in brain innate immune response system and expressed on the membranes of microglia, astrocytes, neurons, and endothelial cells [55]. RAGE is also expressed under normal physiological conditions in neurons, immune cells, activated endothelial cells, glia cells, and vascular smooth muscle cells [56], and activation of RAGE could also induce the expression and secretion of the pro-inflammatory and proapoptotic mediators to work in concert with TLR2, TLR4, and RAGE to exacerbate brain damage [57, 58]. More comprehensive work is warranted and will help to advance our understanding of the pathogenesis of RIBI and provide new strategies for better prevention and control of the disease.

Pregabalin as a novel GABA analogue binding to the α2-δ subunit of VGCC can inhibit synaptic excitability, reduce the release of the excitatory neurotransmitters, and change the electrophysiological activities of neurons, which is mainly used in the treatment of epilepsy and neuralgia [17]. Based on the results of body surface area calculations and available literatures, pregabalin at a dose of 30 mg/kg showed neuroprotective effects in the animal models of spinal cord injury, epilepsy, multiple sclerosis, traumatic brain injury, cerebral ischemia–reperfusion, and diabetic retinopathy [18, 20–22, 34, 59–61]. In this study, we found another new application of pregabalin which could alleviate neuroinflammation and protect neuronal injury in RIBI. We further confirmed that pregabalin alleviated neuroinflammation by inhibiting the cytoplasmic translocation and release of HMGB1 through in vivo and in vitro experiments. As an old drug, it provides new evidence for clinical application, which can accelerate the clinical trials transformation.

How does pregabalin act on neurons and inhibit neuronal HMGB1 cytoplasmic translocation? Studies have shown that CaMKKβ and PKCα play important roles in HMGB1-mediated inflammatory response [62, 63]. Activated CaMKKβ and PKCα can participate in the regulation of HMGB1 cytoplasmic translocation and secretion. As two calcium-dependent enzymes, the premise of CaMKKβ or PKCα activation is due to increased intracellular calcium ion [62]. Pregabalin has been shown to reduce neuropathic pain through its action on VGCC at pre-synaptic site of neurons and the inhibition of synaptic release of glutamate [15, 16]. We therefore speculate that the increased calcium influx in neurons at the acute stage of RIBI may activate CaMKKβ and PKCα, which trigger HMGB1 translocation from nucleus to cytoplasm and its release from neurons. However, the precise molecules by which pregabalin inhibits HMGB1 nuclear translocation in RIBI requires further investigation.

## Conclusions

**Fig. 8:**
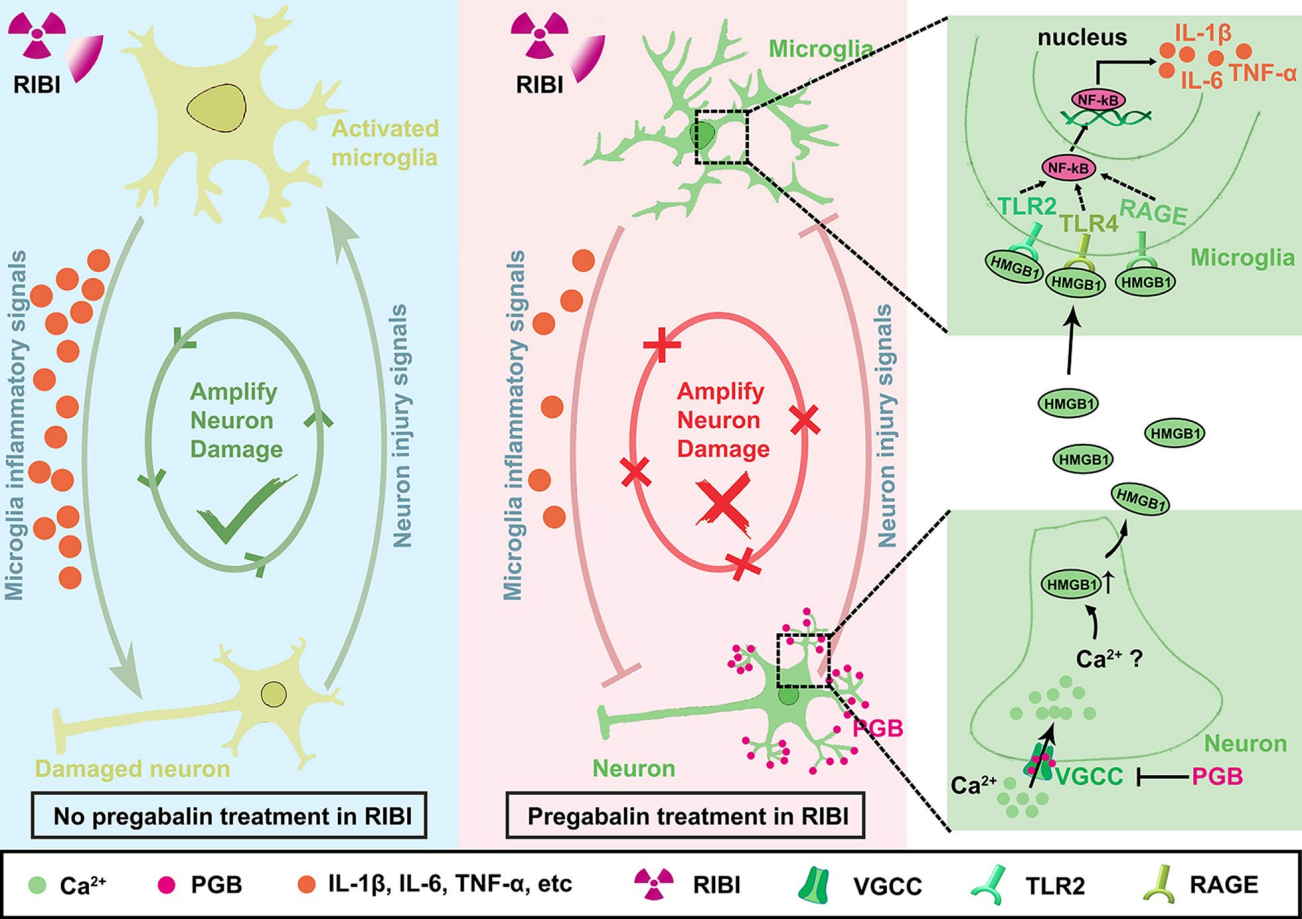
Schematic representation of the mechanistic role of pregabalin in repressing microglia activation and neuronal damage in RIBI

In summary, we provided direct evidence that pregabalin exerted protective effects against RIBI by reducing microglial inflammatory response and preventing neuronal loss via inhibiting HMGB1-TLR2/TLR4/RAGE signaling pathway (Fig. 8). Compared with other strategies, we believe that pregabalin may be a safer and more promising therapeutic strategy for the early intervention of RIBI. In view of the important role of pregabalin in regulating microglial activation and neuroinflammation in RIBI, the potential application of pregabalin in other neurodegenerative diseases is warranted for further investigation.

## Supplementary Information

**Additional file 1: Fig. S1.** Body weight changes in mice receiving radiation or pregabalin. Body weight changes in mice after 14 days of continuous injection of pregabalin (PGB) or saline solution (Con) in RIBI mice. For days 1–4 post-treatment, n = 14–23 mice per group. For days 5–8 post-treatment, n = 10–15 mice per group. For days 9–14 post-treatment, n = 4–9 mice per group. **Fig. S2.** Microglial body size and CD68 expression changes after radiation in vivo. **A** Representative confocal images of IBA1 and CD68 co-labeling in the cortex of mice 3, 7, or 14 days after radiation. Red: IBA1, green: CD68. **B-C** Quantification of the body size of IBA1^+^ cells and the proportion of CD68^+^ area / IBA1^+^ area in the cortex. Data were analyzed by one-way ANOVA followed by the Student’s *t*-test analysis. All other groups were compared with the control group. n = 4 mice per group and 2–3 slices per mouse for immunofluorescence staining. Data were presented as mean ± SEM, *p < 0.05, **p < 0.01, and ***p < 0.001. **Fig. S3**. Pregabalin inhibited microglia activation in the cortex of RIBI mice. **A** Representative images of IBA1 and CD68 co-labeling in the cortex of mice 3 days after radiation. Red: IBA1, green: CD68. **B** Quantification of the proportion of CD68^+^ area / IBA1^+^ area in the cortex of mice 3 days after radiation. **C** Representative images of IBA1 and CD68 co-labeling in the cortex of mice 7 days after radiation. **D** Quantification of the proportion of CD68^+^ area / IBA1^+^ area in the cortex of mice 7 days after radiation. Data were analyzed by one-way ANOVA followed by the Student’s *t*-test analysis. All other groups were compared with the indicated group. n = 4 mice per group and 2–3 slices per mouse for immunofluorescence staining. Data were presented as mean ± SEM, *p < 0.05, **p < 0.01, and ***p < 0.001. **Fig. S4.** Pregabalin inhibited microglia activation in the hippocampus of RIBI mice. **A-B** Representative confocal images of IBA1 and CD68 co-labeling in the hippocampal CA1 (**A**) and DG (**B**) regions of RIBI mice 14 days after pregabalin treatment. Red: IBA1, green: CD68, and blue: DAPI. n = 4 mice per group and 2–3 slices per mouse for immunofluorescence staining. See Fig. 1I-J for statistical data in the main text. **Fig. S5.** Effect of pregabalin on microglial inflammatory response induced by radiation in vitro. **A-D** Q-PCR analysis the effects of pregabalin, with different concentration (1 µM, 6.25 µM, 12.5 µM, 25 µM, and 50 µM), on the mRNA levels of inflammatory factors *Il-1β*, *Tnf-α*, *Cox-2*, and *iNos* in BV2 cells after a single dose of 10 Gy radiation. Data were analyzed by one-way ANOVA followed by the Student’s *t*-test analysis. All other groups were compared with the indicated group. n = 3 per group for Q-PCR analysis in vitro. Data were presented as mean ± SEM, ns = not significant, **p < 0.01, and ***p < 0.001. **Fig. S6.** Effect of pregabalin on IL-6 and TNF-α expressions in microglia after radiation. **A** Representative immunofluorescent images of IL-6 and β-tubulin in BV2 cells among the different groups. Staining with β-tubulin to visualize cytoskeleton and staining with DAPI to visualize nucleus. **B** The fluorescence intensity data of IL-6 were recorded by confocal microscopy. **C** Representative immunofluorescent images of TNF-α and β-tubulin in BV2 cells among the different groups. **D** The fluorescence intensity data of TNF-α were recorded by confocal microscopy. Data were analyzed by one-way ANOVA followed by the Student’s *t*-test analysis. All other groups were compared with the indicated group. n = 3 per group for immunofluorescence staining in vitro. Data were presented as mean ± SEM, ns = not significant and **p < 0.01. **Fig. S7.** Pregabalin inhibited microglial inflammatory response not by acting on astrocyte in vitro. **A** Schematic diagram of BV2 cells incubated with the culture supernatant from astrocyte after different treatment. **B-E** Q-PCR analysis of *Il-1β*, *Tnf-α*, *iNos*, and *Icam-1* mRNA levels in BV2 cells after incubated with the supernatant from pregabalin-treated astrocyte. Data were analyzed by one-way ANOVA followed by the Student’s *t*-test analysis. All other groups were compared with the indicated group. n = 4 per group for Q-PCR analysis in vitro. Data were presented as mean ± SEM, ns = not significant and *p < 0.05. **Fig. S8.** Effect of pregabalin on potential chemokines in injured neurons. **A-G** Q-PCR analysis of *Mmp9*, *Cgrp*, *Tac1*, *Cx3cl1*, *Ccl21*, *Mmp2*, and *Ccl2* mRNA levels in neurons after treatment with the different supernatant from BV2 cells. Data were analyzed by one-way ANOVA followed by the Student’s *t*-test analysis. All other groups were compared with the indicated group. n = 3 per group for Q-PCR analysis in vitro. Data were presented as mean ± SEM, ns = not significant, *p < 0.05, and ***p < 0.001. **Fig. S9.** Knocking out TLR2/TLR4/RAGE mitigated microglia activation. **A-C** Schematic diagram of CRISPR/Cas9-mediated TLR2/TLR4/RAGE knockout in BV2 cells and Q-PCR analysis was used to detect the knockout efficiency. **D-F** Q-PCR analysis of *Il-6*, *Tnf-α*, and *Cox-2* mRNA levels in activated BV2 cells which were treated with culture supernatant from radiation-injured (activated) or normal (control) neurons for 24 h. Data were analyzed by one-way ANOVA followed by the Student’s *t*-test analysis. All other groups were compared with the indicated group. n = 3–4 per group for Q-PCR analysis in vitro. Data were presented as mean ± SEM, *p < 0.05, **p < 0.01, and ***p < 0.001. **Table S1.** List of primers used for RNA analyses. **Table S2.** List of antibodies used in this study. **Table S3.** List of gRNA sequences used for CRISPR/Cas9-mediated gene knockout. **Table S4.** List of oligonucleotide sequences used for plasmid construction. **Table S5.** List of primers used for the verification of CRISPR/Cas9-mediated gene knockout.

## Data Availability

All data supporting our results are available from the corresponding authors upon reasonable request.

## Abbreviations

RIBI: Radiation-induced brain injury
CNSL: Central nervous system
VEGF: Vascular endothelial growth factor
VGCC: Voltage-gated calcium channels
BBB: Blood–brain barrier
GABA: Gamma-aminobutyric acid
HMGB1: High-mobility group box 1
Q-PCR: Quantitative real-time polymerase chain reaction
Q-PCR: Quantitative real-time polymerase chain reaction
TUNEL: Terminal deoxynucleotidyl transferase dUTP Nick-end labeling
ELISA: Enzyme-linked immunosorbent assay
TBI: Traumatic brain injury

## References

[1] Owonikoko TK, Arbiser J, Zelnak A, Shu HK, Shim H, Robin AM, Kalkanis SN, Whitsett TG, Salhia B, Tran NL (2014). Current approaches to the treatment of metastatic brain tumours. Nat Rev Clin Oncol.

[2] Crossen JR, Garwood D, Glatstein E, Neuwelt EA (1994). Neurobehavioral sequelae of cranial irradiation in adults: a review of radiation-induced encephalopathy. J Clin Oncol.

[3] Cheung MC, Chan AS, Law SC, Chan JH, Tse VK (2003). Impact of radionecrosis on cognitive dysfunction in patients after radiotherapy for nasopharyngeal carcinoma. Cancer.

[4] He B, Wang X, He Y, Li H, Yang Y, Shi Z, Liu Q, Wu M, Sun H, Xie J (2020). Gamma ray-induced glial activation and neuronal loss occur before the delayed onset of brain necrosis. FASEB J.

[5] Wilke C, Grosshans D, Duman J, Brown P, Li J (2018). Radiation-induced cognitive toxicity: pathophysiology and interventions to reduce toxicity in adults. Neuro Oncol.

[6] Robbins M, Greene-Schloesser D, Peiffer A, Shaw E, Chan M, Wheeler K (2012). Radiation-induced brain injury: A review. Front Oncol.

[7] Jeyaretna DS, Curry WT, Batchelor TT, Stemmer-Rachamimov A, Plotkin SR (2011). Exacerbation of cerebral radiation necrosis by bevacizumab. J Clin Oncol.

[8] Li Y, Huang X, Jiang J, Hu W, Hu J, Cai J, Rong X, Cheng J, Xu Y, Wu R (2018). Clinical variables for prediction of the therapeutic effects of bevacizumab monotherapy in nasopharyngeal carcinoma patients with radiation-induced brain necrosis. Int J Radiat Oncol Biol Phys.

[9] Lumniczky K, Szatmari T, Safrany G (2017). Ionizing Radiation-Induced Immune and Inflammatory Reactions in the Brain. Front Immunol.

[10] Greene-Schloesser D, Robbins ME, Peiffer AM, Shaw EG, Wheeler KT, Chan MD (2012). Radiation-induced brain injury: A review. Front Oncol.

[11] Salter MW, Stevens B (2017). Microglia emerge as central players in brain disease. Nat Med.

[12] Woodburn SC, Bollinger JL, Wohleb ES (2021). The semantics of microglia activation: neuroinflammation, homeostasis, and stress. J Neuroinflammation.

[13] Peng Y, Lu K, Li Z, Zhao Y, Wang Y, Hu B, Xu P, Shi X, Zhou B, Pennington M, et al: Blockade of Kv1.3 channels ameliorates radiation-induced brain injury. Neuro Oncol 2014, 16:528–539. 10.1093/neuonc/not221 PMC3956348 24305723

[14] Jenrow KA, Brown SL, Lapanowski K, Naei H, Kolozsvary A, Kim JH (2013). Selective inhibition of microglia-mediated neuroinflammation mitigates radiation-induced cognitive impairment. Radiat Res.

[15] Taylor CP, Angelotti T, Fauman E (2007). Pharmacology and mechanism of action of pregabalin: the calcium channel alpha2-delta (alpha2-delta) subunit as a target for antiepileptic drug discovery. Epilepsy Res.

[16] Field MJ, Cox PJ, Stott E, Melrose H, Offord J, Su TZ, Bramwell S, Corradini L, England S, Winks J (2006). Identification of the alpha2-delta-1 subunit of voltage-dependent calcium channels as a molecular target for pain mediating the analgesic actions of pregabalin. Proc Natl Acad Sci U S A.

[17] Toth C (2014). Pregabalin: latest safety evidence and clinical implications for the management of neuropathic pain. Ther Adv Drug Saf.

[18] Ha KY, Kim YH, Rhyu KW, Kwon SE (2008). Pregabalin as a neuroprotector after spinal cord injury in rats. Eur Spine J.

[19] Recla JM, Sarantopoulos CD (2009). Combined use of pregabalin and memantine in fibromyalgia syndrome treatment: a novel analgesic and neuroprotective strategy?. Med Hypotheses.

[20] Ali SA, Zaitone SA, Dessouki AA, Ali AA (2019). Pregabalin affords retinal neuroprotection in diabetic rats: Suppression of retinal glutamate, microglia cell expression and apoptotic cell death. Exp Eye Res.

[21] Hundehege P, Fernandez-Orth J, Romer P, Ruck T, Muntefering T, Eichler S, Cerina M, Epping L, Albrecht S, Menke AF (2018). Targeting voltage-dependent calcium channels with pregabalin exerts a direct neuroprotective effect in an animal model of multiple sclerosis. Neurosignals.

[22] Shamsi Meymandi M, Soltani Z, Sepehri G, Amiresmaili S, Farahani F, Moeini Aghtaei M (2018). Effects of pregabalin on brain edema, neurologic and histologic outcomes in experimental traumatic brain injury. Brain Res Bull.

[23] Asci S, Demirci S, Asci H, Doguc DK, Onaran I (2016). Neuroprotective effects of pregabalin on cerebral ischemia and reperfusion. Balkan Med J.

[24] Jiang J, Li Y, Shen Q, Rong X, Huang X, Li H, Zhou L, Mai HQ, Zheng D, Chen MY (2019). Effect of pregabalin on radiotherapy-related neuropathic pain in patients with head and neck cancer: a randomized controlled trial. J Clin Oncol.

[25] Sun Q, Wu W, Hu YC, Li H, Zhang D, Li S, Li W, Li WD, Ma B, Zhu JH (2014). Early release of high-mobility group box 1 (HMGB1) from neurons in experimental subarachnoid hemorrhage in vivo and in vitro. J Neuroinflammation.

[26] Xu P, Xu Y, Hu B, Wang J, Pan R, Murugan M, Wu LJ, Tang Y (2015). Extracellular ATP enhances radiation-induced brain injury through microglial activation and paracrine signaling via P2X7 receptor. Brain Behav Immun.

[27] Zhang Z, Shen Q, Wu X, Zhang D, Xing D (2020). Activation of PKA/SIRT1 signaling pathway by photobiomodulation therapy reduces Abeta levels in Alzheimer's disease models. Aging Cell.

[28] Stemmer M, Thumberger T, Del Sol KM, Wittbrodt J, Mateo JL (2015). CCTop: An Intuitive, Flexible and Reliable CRISPR/Cas9 Target Prediction Tool. PLoS ONE.

[29] Milano MT, Grimm J, Niemierko A, Soltys SG, Moiseenko V, Redmond KJ, Yorke E, Sahgal A, Xue J, Mahadevan A (2021). Single- and Multifraction Stereotactic Radiosurgery Dose/Volume Tolerances of the Brain. Int J Radiat Oncol Biol Phys.

[30] Yang L, Yang J, Li G, Li Y, Wu R, Cheng J, Tang Y (2017). Pathophysiological Responses in Rat and Mouse Models of Radiation-Induced Brain Injury. Mol Neurobiol.

[31] Liu Y, Xiao S, Liu J, Zhou H, Liu Z, Xin Y, Suo WZ (2010). An experimental study of acute radiation-induced cognitive dysfunction in a young rat model. AJNR Am J Neuroradiol.

[32] Gaber MW, Sabek OM, Fukatsu K, Wilcox HG, Kiani MF, Merchant TE (2003). Differences in ICAM-1 and TNF-alpha expression between large single fraction and fractionated irradiation in mouse brain. Int J Radiat Biol.

[33] Xu Y, Hu W, Liu Y, Xu P, Li Z, Wu R, Shi X, Tang Y (2016). P2Y6 Receptor-Mediated Microglial Phagocytosis in Radiation-Induced Brain Injury. Mol Neurobiol.

[34] Nair AB, Jacob S (2016). A simple practice guide for dose conversion between animals and human. J Basic Clin Pharm.

[35] Leavitt RJ, Acharya MM, Baulch JE, Limoli CL (2020). Extracellular Vesicle-Derived miR-124 Resolves Radiation-Induced Brain Injury. Cancer Res.

[36] Newcombe EA, Camats-Perna J, Silva ML, Valmas N, Huat TJ, Medeiros R (2018). Inflammation: the link between comorbidities, genetics, and Alzheimer's disease. J Neuroinflammation.

[37] Block ML, Zecca L, Hong JS (2007). Microglia-mediated neurotoxicity: uncovering the molecular mechanisms. Nat Rev Neurosci.

[38] Abu-Rish EY, Mansour AT, Mansour HT, Dahabiyeh LA, Aleidi SM, Bustanji Y (2020). Pregabalin inhibits in vivo and in vitro cytokine secretion and attenuates spleen inflammation in Lipopolysaccharide/Concanavalin A -induced murine models of inflammation. Sci Rep.

[39] Park S, Ahn ES, Han DW, Lee JH, Min KT, Kim H, Hong YW (2008). Pregabalin and gabapentin inhibit substance P-induced NF-kappaB activation in neuroblastoma and glioma cells. J Cell Biochem.

[40] Kirkley KS, Popichak KA, Afzali MF, Legare ME, Tjalkens RB (2017). Microglia amplify inflammatory activation of astrocytes in manganese neurotoxicity. J Neuroinflammation.

[41] Tian L, Ma L, Kaarela T, Li Z (2012). Neuroimmune crosstalk in the central nervous system and its significance for neurological diseases. J Neuroinflammation.

[42] Posfai B, Cserep C, Orsolits B, Denes A (2019). New Insights into Microglia-Neuron Interactions: A Neuron's Perspective. Neuroscience.

[43] Fang P, Schachner M, Shen YQ (2012). HMGB1 in development and diseases of the central nervous system. Mol Neurobiol.

[44] Yang H, Hreggvidsdottir HS, Palmblad K, Wang H, Ochani M, Li J, Lu B, Chavan S, Rosas-Ballina M, Al-Abed Y (2010). A critical cysteine is required for HMGB1 binding to Toll-like receptor 4 and activation of macrophage cytokine release. Proc Natl Acad Sci U S A.

[45] Takizawa T, Shibata M, Kayama Y, Shimizu T, Toriumi H, Ebine T, Unekawa M, Koh A, Yoshimura A, Suzuki N (2017). High-mobility group box 1 is an important mediator of microglial activation induced by cortical spreading depression. J Cereb Blood Flow Metab.

[46] Fan H, Tang HB, Chen Z, Wang HQ, Zhang L, Jiang Y, Li T, Yang CF, Wang XY, Li X (2020). Inhibiting HMGB1-RAGE axis prevents pro-inflammatory macrophages/microglia polarization and affords neuroprotection after spinal cord injury. J Neuroinflammation.

[47] Turnquist C, Harris BT, Harris CC (2020). Radiation-induced brain injury: current concepts and therapeutic strategies targeting neuroinflammation. Neurooncol Adv.

[48] Xu L, He D, Bai Y (2016). Microglia-Mediated Inflammation and Neurodegenerative Disease. Mol Neurobiol.

[49] Hickman S, Izzy S, Sen P, Morsett L, El Khoury J (2018). Microglia in neurodegeneration. Nat Neurosci.

[50] Kettenmann H, Kirchhoff F, Verkhratsky A (2013). Microglia: new roles for the synaptic stripper. Neuron.

[51] Paudel YN, Shaikh MF, Chakraborti A, Kumari Y, Aledo-Serrano A, Aleksovska K, Alvim MKM, Othman I (2018). HMGB1: A Common Biomarker and Potential Target for TBI, neuroinflammation, epilepsy, and cognitive dysfunction. Front Neurosci.

[52] Wang L, Zhang J, Wang B, Wang G, Xu J (2015). Blocking HMGB1 signal pathway protects early radiation-induced lung injury. Int J Clin Exp Pathol.

[53] Yang H, Zeng Q, Silverman HA (2021). HMGB1 released from nociceptors mediates inflammation. Proc Natl Acad Sci U S A.

[54] Faraco G, Fossati S, Bianchi ME, Patrone M, Pedrazzi M, Sparatore B, Moroni F, Chiarugi A (2007). High mobility group box 1 protein is released by neural cells upon different stresses and worsens ischemic neurodegeneration in vitro and in vivo. J Neurochem.

[55] Wang Y, Ge P, Yang L, Wu C, Zha H, Luo T, Zhu Y (2014). Protection of ischemic post conditioning against transient focal ischemia-induced brain damage is associated with inhibition of neuroinflammation via modulation of TLR2 and TLR4 pathways. J Neuroinflammation.

[56] Juranek J, Mukherjee K, Kordas B (2022). Role of RAGE in the Pathogenesis of Neurological Disorders. Neurosci Bull.

[57] Choi BR, Cho WH, Kim J, Lee HJ, Chung C, Jeon WK, Han JS (2014). Increased expression of the receptor for advanced glycation end products in neurons and astrocytes in a triple transgenic mouse model of Alzheimer's disease. Exp Mol Med.

[58] Rosenberger K, Derkow K, Dembny P, Krüger C, Schott E, Lehnardt S (2014). The impact of single and pairwise Toll-like receptor activation on neuroinflammation and neurodegeneration. J Neuroinflammation.

[59] Daneshdoust D, Khalili-Fomeshi M, Ghasemi-Kasman M, Ghorbanian D, Hashemian M, Gholami M, Moghadamnia A, Shojaei A (2017). Pregabalin enhances myelin repair and attenuates glial activation in lysolecithin-induced demyelination model of rat optic chiasm. Neuroscience.

[60] Qureshi IH, Riaz A, Khan RA, Siddiqui AA (2017). Synergistic anticonvulsant effects of pregabalin and amlodipine on acute seizure model of epilepsy in mice. Metab Brain Dis.

[61] Silva GA, Pradella F, Moraes A, Farias A, dos Santos LM, de Oliveira AL (2014). Impact of pregabalin treatment on synaptic plasticity and glial reactivity during the course of experimental autoimmune encephalomyelitis. Brain Behav.

[62] Zhao P, Ye T, Yan X, Hu X, Liu P, Wang X (2017). HMGB1 release by H2O2-induced hepatocytes is regulated through calcium overload and 58-F interference. Cell Death Discov.

[63] Tsung A, Klune JR, Zhang X, Jeyabalan G, Cao Z, Peng X, Stolz DB, Geller DA, Rosengart MR, Billiar TR (2007). HMGB1 release induced by liver ischemia involves Toll-like receptor 4 dependent reactive oxygen species production and calcium-mediated signaling. J Exp Med.

