# Supplementary material for: Pregabalin mitigates microglial activation and neuronal injury by inhibiting HMGB1 signaling pathway in radiation-induced brain injury
Source: J Neuroinflammation. 2022 Sep 21;19:231. doi: 10.1186/s12974-022-02596-7 (PMC9490947; doi:10.1186/s12974-022-02596-7)
Supplement: Supplementary file 1 — Additional file 1: Fig. S1. Body weight changes in mice receiving radiation or pregabalin. Body weight changes in mice after 14 days of continuous injection of pregabalin (PGB) or saline solution (Con) in RIBI mice. For days 1–4 post-treatment, n = 14–23 mice per group. For days 5–8 post-treatment, n = 10–15 mice per group. For days 9–14 post-treatment, n = 4–9 mice per group. Fig. S2. Microglial body size and CD68 expression changes after radiation in vivo. A Representative confocal images of IBA1 and CD68 co-labeling in the cortex of mice 3, 7, or 14 days after radiation. Red: IBA1, green: CD68. B-C Quantification of the body size of IBA1+ cells and the proportion of CD68+ area / IBA1+ area in the cortex. Data were analyzed by one-way ANOVA followed by the Student’s t-test analysis. All other groups were compared with the control group. n = 4 mice per group and 2–3 slices per mouse for immunofluorescence staining. Data were presented as mean ± SEM, *p < 0.05, **p < 0.01, and ***p < 0.001. Fig. S3. Pregabalin inhibited microglia activation in the cortex of RIBI mice. A Representative images of IBA1 and CD68 co-labeling in the cortex of mice 3 days after radiation. Red: IBA1, green: CD68. B Quantification of the proportion of CD68+ area / IBA1+ area in the cortex of mice 3 days after radiation. C Representative images of IBA1 and CD68 co-labeling in the cortex of mice 7 days after radiation. D Quantification of the proportion of CD68+ area / IBA1+ area in the cortex of mice 7 days after radiation. Data were analyzed by one-way ANOVA followed by the Student’s t-test analysis. All other groups were compared with the indicated group. n = 4 mice per group and 2–3 slices per mouse for immunofluorescence staining. Data were presented as mean ± SEM, *p < 0.05, **p < 0.01, and ***p < 0.001. Fig. S4. Pregabalin inhibited microglia activation in the hippocampus of RIBI mice. A-B Representative confocal images of IBA1 and CD68 co-labeling in the hippocampal CA1 (A) and DG (B) [file 12974_2022_2596_MOESM1_ESM.docx]

**Supplementary Information**

**Supplementary Figures**

**Figure S1. Body weight changes in mice receiving radiation or pregabalin.**


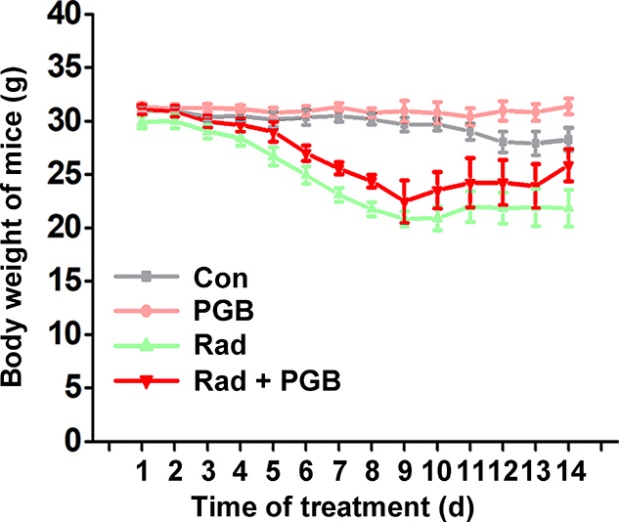


**Fig. S1** Body weight changes in mice receiving radiation or pregabalin. Body weight changes in mice after 14 days of continuous injection of pregabalin (PGB) or saline solution (Con) in RIBI mice. For days 1-4 post-treatment, n = 14-23 mice per group. For days 5-8 post-treatment, n = 10-15 mice per group. For days 9-14 post-treatment, n = 4-9 mice per group.

**Figure S2. Microglial body size and CD68 expression changes after radiation *in vivo*.**


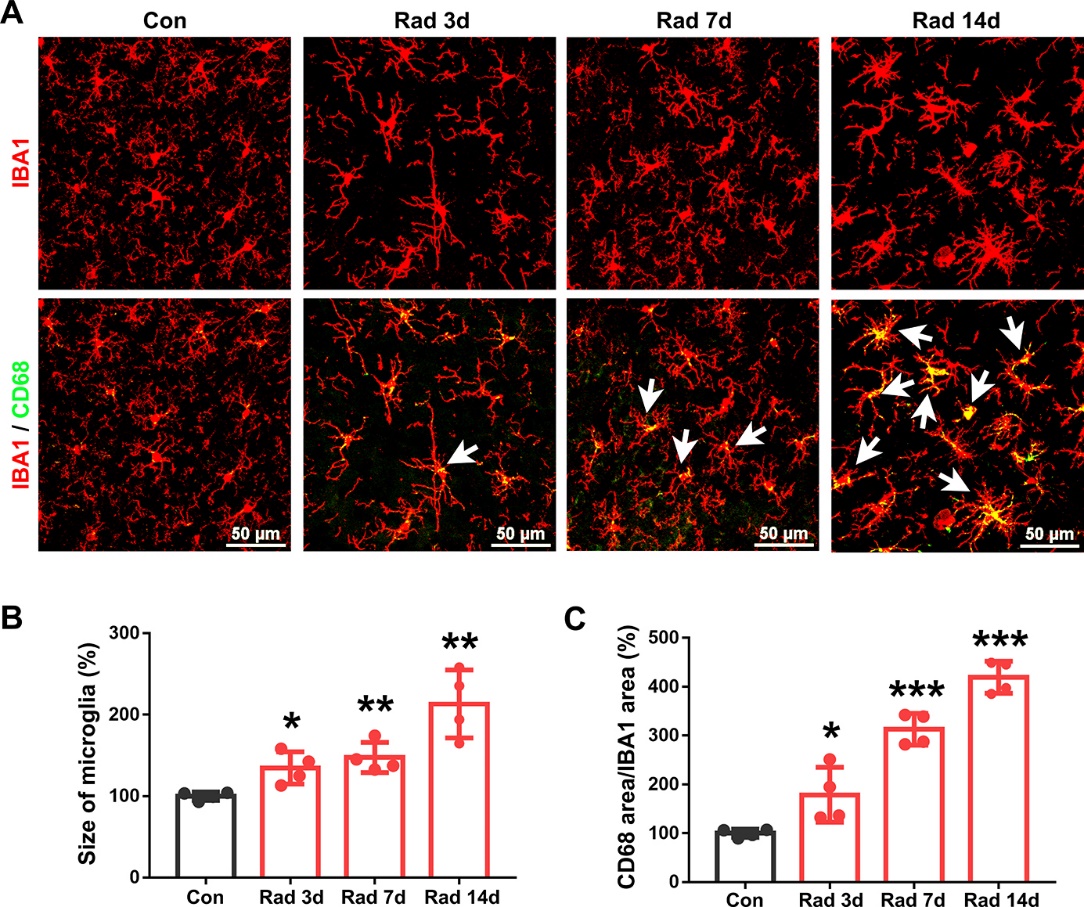


**Fig. S2** Microglial body size and CD68 expression changes after radiation *in vivo*. **A** Representative confocal images of IBA1 and CD68 co-labeling in the cortex of mice 3, 7, or 14 days after radiation. Red: IBA1, green: CD68. **B-C** Quantification of the body size of IBA1^+^ cells and the proportion of CD68^+^ area / IBA1^+^ area in the cortex. Data were analyzed by one-way ANOVA followed by the Student’s *t*-test analysis. All other groups were compared with the control group. n = 4 mice per group and 2–3 slices per mouse for immunofluorescence staining. Data were presented as mean ± SEM, *p < 0.05, **p < 0.01, and ***p < 0.001.

**Figure S3. Pregabalin inhibited microglia activation in the cortex of RIBI mice.**


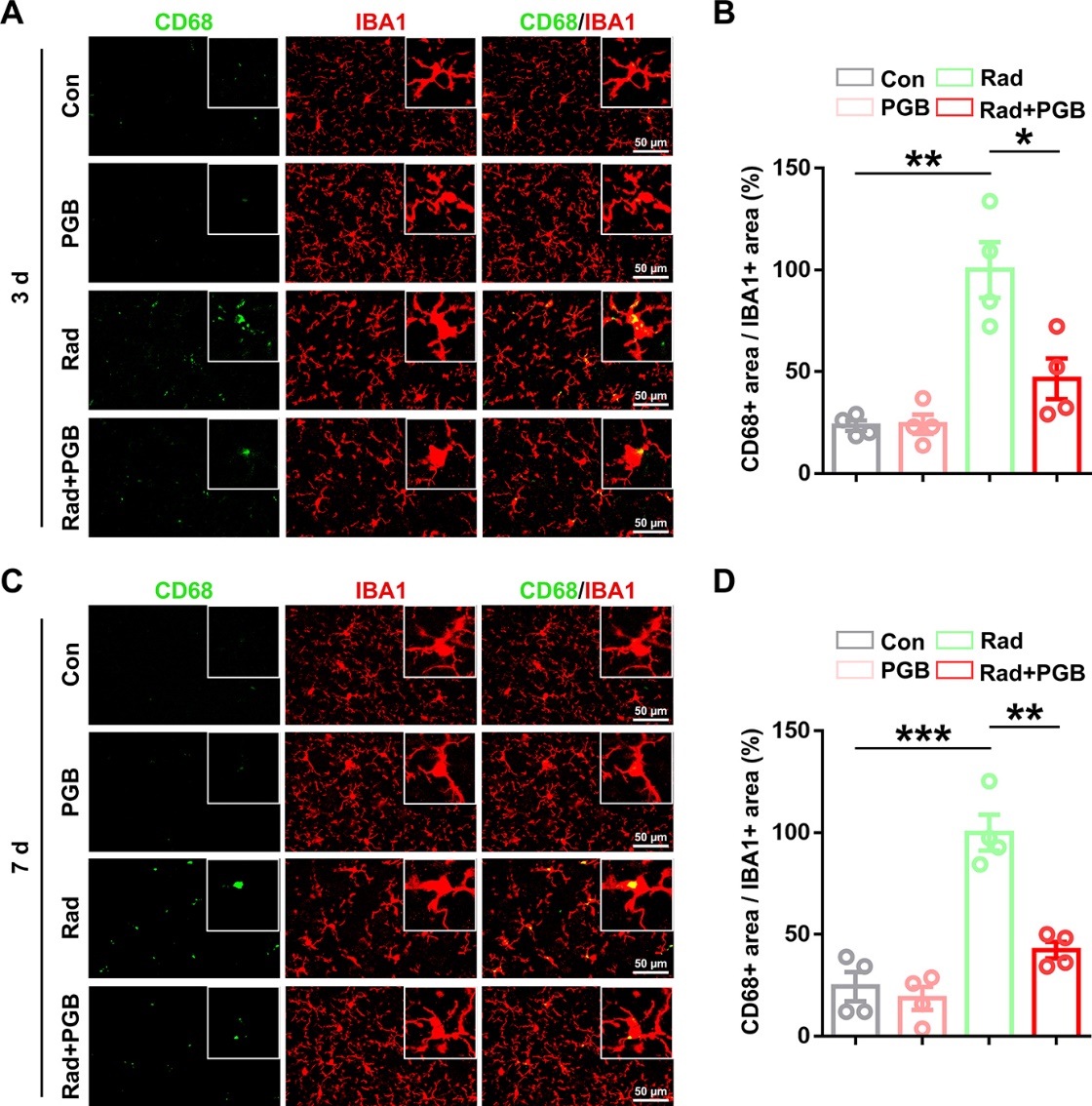


**Fig. S3**. Pregabalin inhibited microglia activation in the cortex of RIBI mice. **A** Representative images of IBA1 and CD68 co-labeling in the cortex of mice 3 days after radiation. Red: IBA1, green: CD68. **B** Quantification of the proportion of CD68^+^ area / IBA1^+^ area in the cortex of mice 3 days after radiation. **C** Representative images of IBA1 and CD68 co-labeling in the cortex of mice 7 days after radiation. **D** Quantification of the proportion of CD68^+^ area / IBA1^+^ area in the cortex of mice 7 days after radiation. Data were analyzed by one-way ANOVA followed by the Student’s *t*-test analysis. All other groups were compared with the indicated group. n = 4 mice per group and 2–3 slices per mouse for immunofluorescence staining. Data were presented as mean ± SEM, *p < 0.05, **p < 0.01, and ***p < 0.001.

**Figure S4. Pregabalin inhibited microglia activation in the hippocampus of RIBI mice.**


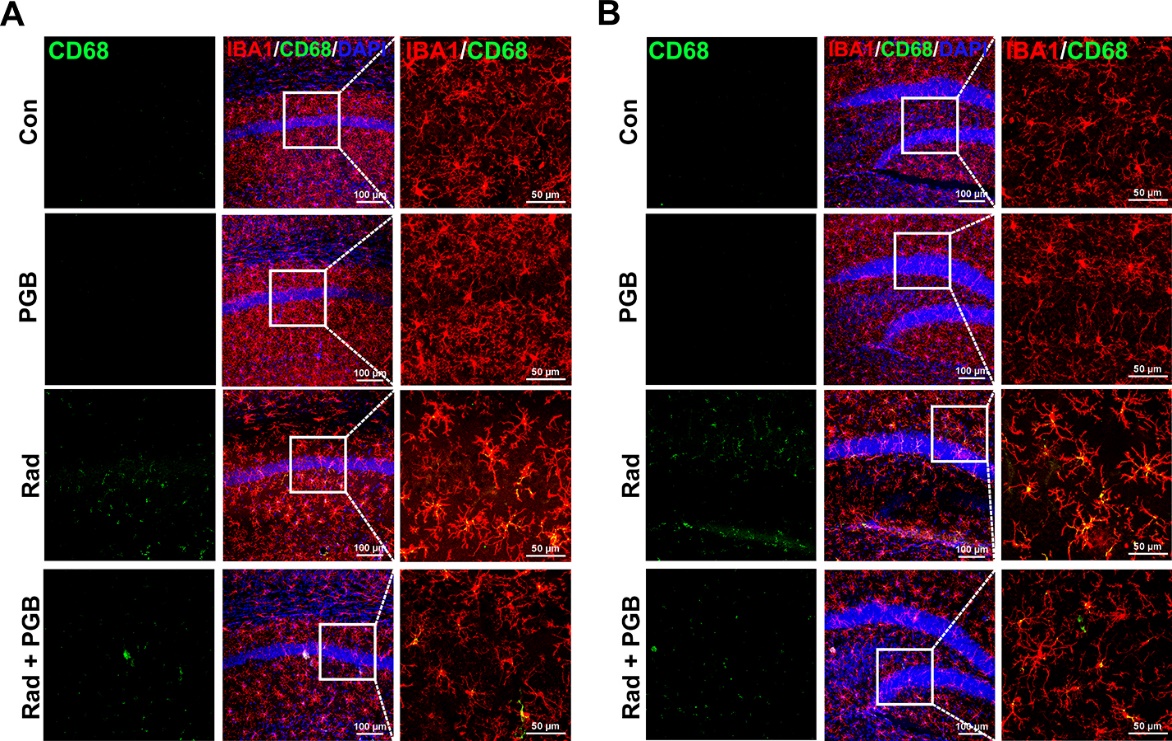


**Fig. S4** Pregabalin inhibited microglia activation in the hippocampus of RIBI mice. **A-B** Representative confocal images of IBA1 and CD68 co-labeling in the hippocampal CA1 (**A**) and DG (**B**) regions of RIBI mice 14 days after pregabalin treatment. Red: IBA1, green: CD68, and blue: DAPI. n = 4 mice per group and 2–3 slices per mouse for immunofluorescence staining. See Fig. 1I-J for statistical data in the main text.

**Figure S5. Effect of pregabalin on microglial inflammatory response induced by radiation *in vitro*.**


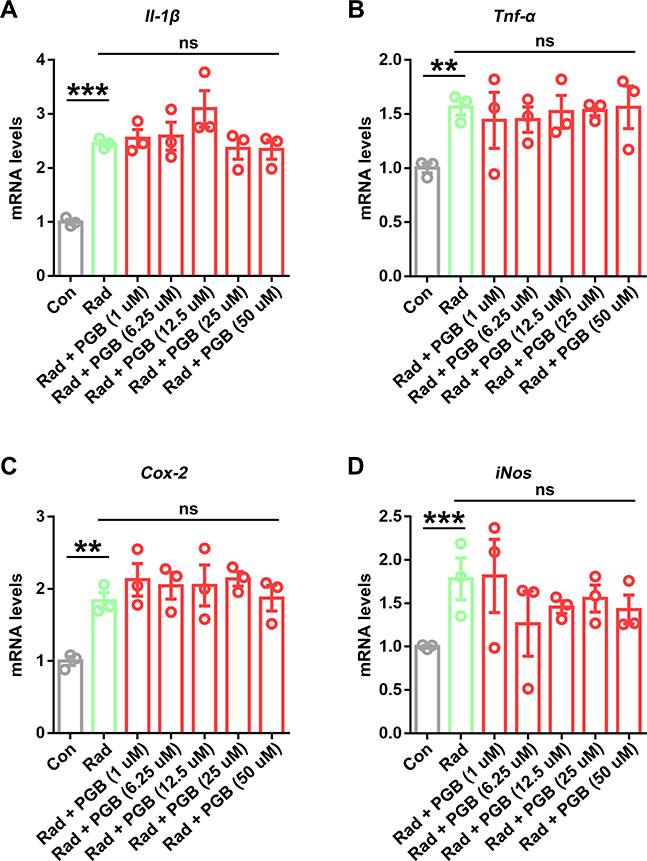


**Fig. S5** Effect of pregabalin on microglial inflammatory response induced by radiation *in vitro*. **A-D** Q-PCR analysis the effects of pregabalin, with different concentration (1 µM, 6.25 µM, 12.5 µM, 25 µM, and 50 µM), on the mRNA levels of inflammatory factors *Il-1β*, *Tnf-α*, *Cox-2*, and *iNos* in BV2 cells after a single dose of 10 Gy radiation. Data were analyzed by one-way ANOVA followed by the Student’s *t*-test analysis. All other groups were compared with the indicated group. n = 3 per group for Q-PCR analysis *in vitro*. Data were presented as mean ± SEM, ns = not significant, **p < 0.01, and ***p < 0.001.

**Figure S6. Effect of pregabalin on IL-6 and TNF-α expressions in microglia after radiation.**


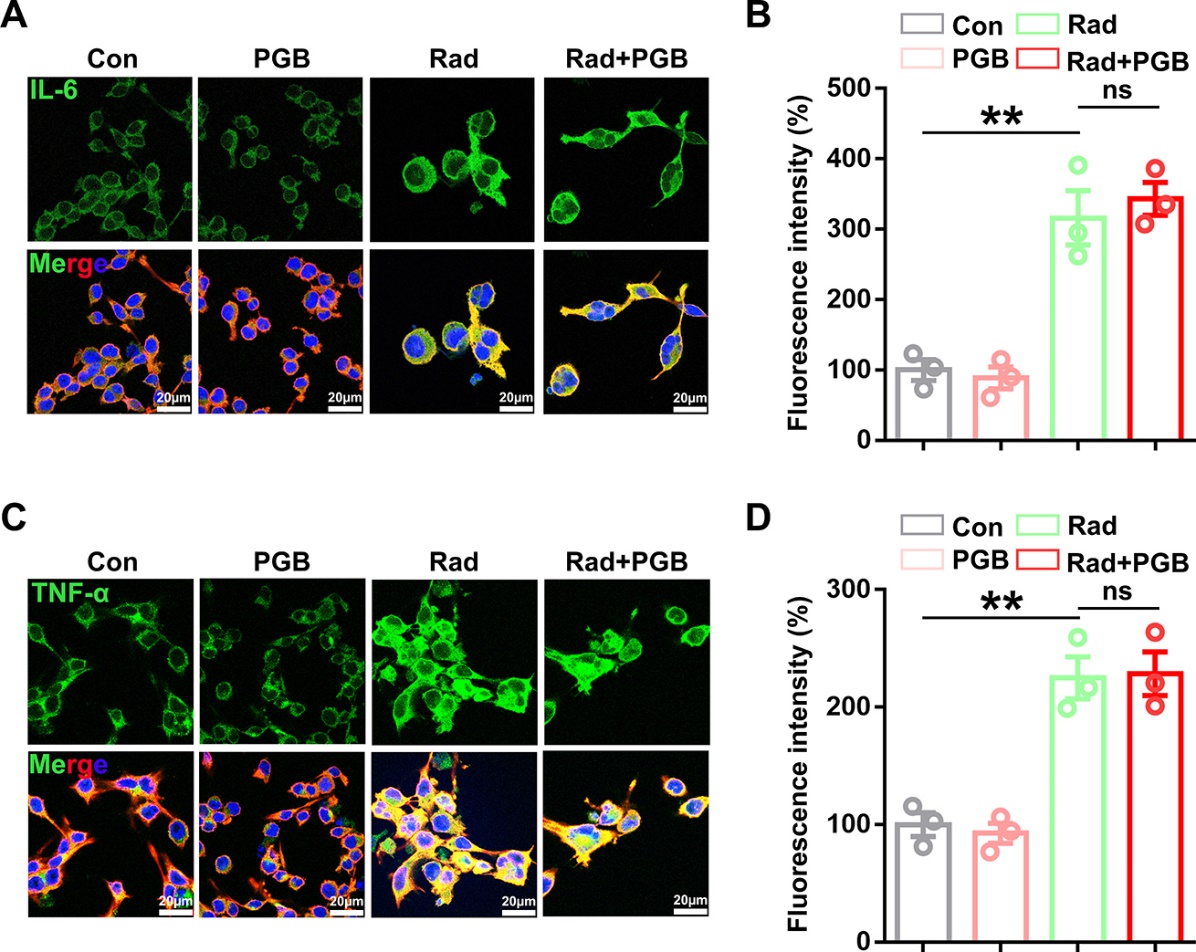


**Fig. S6** Effect of pregabalin on IL-6 and TNF-α expressions in microglia after radiation. **A** Representative immunofluorescent images of IL-6 and β-tubulin in BV2 cells among the different groups. Staining with β-tubulin to visualize cytoskeleton and staining with DAPI to visualize nucleus. **B** The fluorescence intensity data of IL-6 were recorded by confocal microscopy. **C** Representative immunofluorescent images of TNF-α and β-tubulin in BV2 cells among the different groups. **D** The fluorescence intensity data of TNF-α were recorded by confocal microscopy. Data were analyzed by one-way ANOVA followed by the Student’s *t*-test analysis. All other groups were compared with the indicated group. n = 3 per group for immunofluorescence staining *in vitro*. Data were presented as mean ± SEM, ns = not significant and **p < 0.01.

**Figure S7. Pregabalin inhibited microglial inflammatory response not by acting on astrocyte in vitro.**


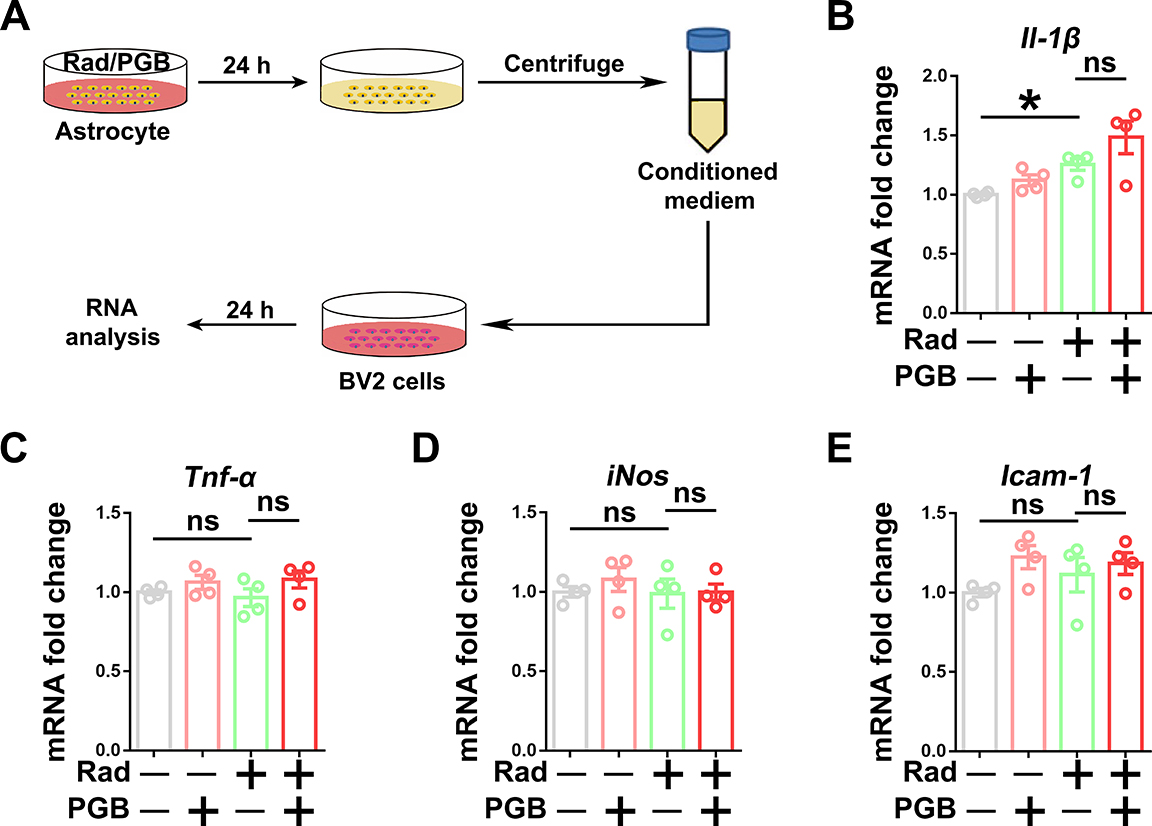


**Fig. S7** Pregabalin inhibited microglial inflammatory response not by acting on astrocyte *in vitro*. **A** Schematic diagram of BV2 cells incubated with the culture supernatant from astrocyte after different treatment. **B-E** Q-PCR analysis of *Il-1β*, *Tnf-α*, *iNos*, and *Icam-1* mRNA levels in BV2 cells after incubated with the supernatant from pregabalin-treated astrocyte. Data were analyzed by one-way ANOVA followed by the Student’s *t*-test analysis. All other groups were compared with the indicated group. n = 4 per group for Q-PCR analysis *in vitro*. Data were presented as mean ± SEM, ns = not significant and *p < 0.05.

**Figure S8. Effect of pregabalin on potential chemokines in injured neurons.**


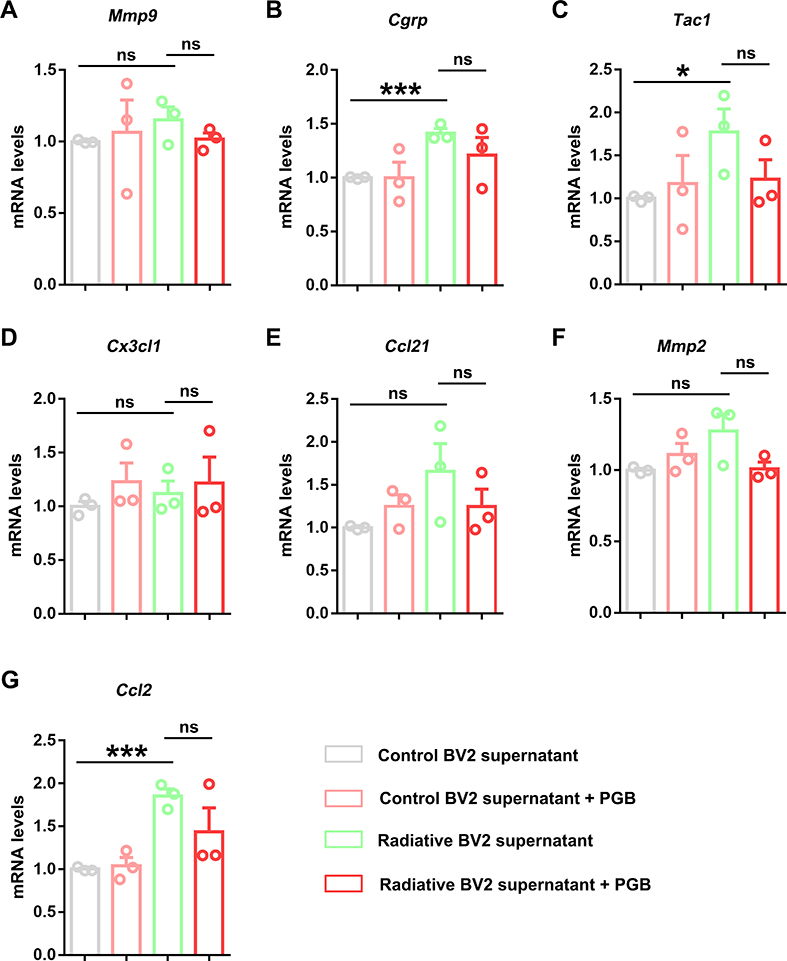


**Fig. S8** Effect of pregabalin on potential chemokines in injured neurons. **A-G** Q-PCR analysis of *Mmp9*, *Cgrp*, *Tac1*, *Cx3cl1*, *Ccl21*, *Mmp2*, and *Ccl2* mRNA levels in neurons after treatment with the different supernatant from BV2 cells. Data were analyzed by one-way ANOVA followed by the Student’s *t*-test analysis. All other groups were compared with the indicated group. n = 3 per group for Q-PCR analysis *in vitro*. Data were presented as mean ± SEM, ns = not significant, *p < 0.05, and ***p < 0.001.

**Figure S9. Knocking out TLR2/TLR4/RAGE mitigated microglia activation.**


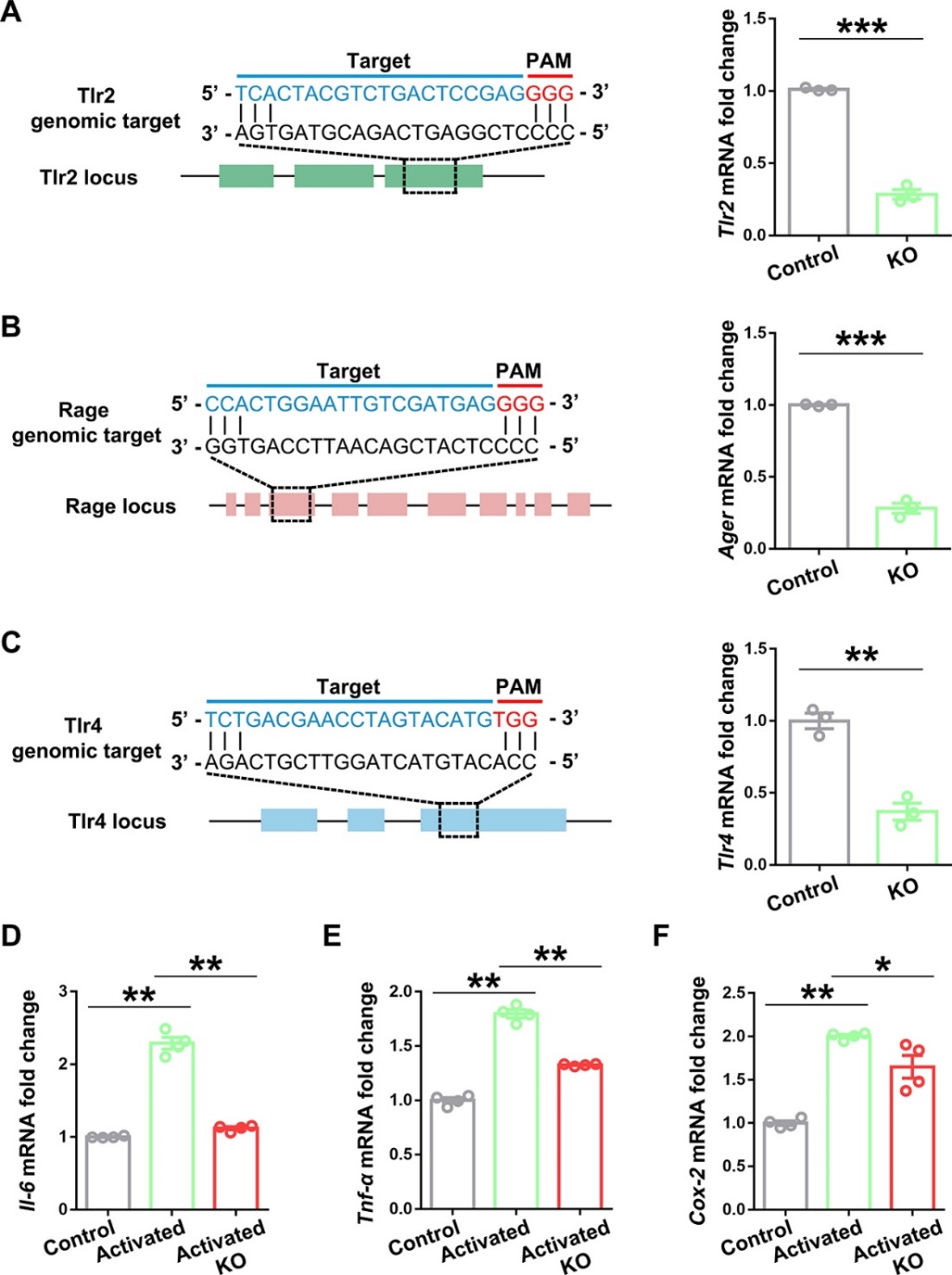


**Fig. S9** Knocking out TLR2/TLR4/RAGE mitigated microglia activation. **A-C** Schematic diagram of CRISPR/Cas9-mediated TLR2/TLR4/RAGE knockout in BV2 cells and Q-PCR analysis was used to detect the knockout efficiency. **D-F** Q-PCR analysis of *Il-6*, *Tnf-α*, and *Cox-2* mRNA levels in activated BV2 cells which were treated with culture supernatant from radiation-injured (activated) or normal (control) neurons for 24 h. Data were analyzed by one-way ANOVA followed by the Student’s *t*-test analysis. All other groups were compared with the indicated group. n = 3-4 per group for Q-PCR analysis *in vitro*. Data were presented as mean ± SEM, *p < 0.05, **p < 0.01, and ***p < 0.001.

**Supplementary** **Tables**

**Table S1. List of primers used for RNA analyses.**

| RT-PCR primers Sequence |
| --- |
| *Il-1β* Forward Primer GCAACTGTTCCTGAACTCAACT  Reverse Primer ATCTTTTGGGGTCCGTCAACT  *Il-6* Forward Primer TAGTCCTTCCTACCCCAATTTCC  Reverse Primer TTGGTCCTTAGCCACTCCTTC  *Tnf-α* Forward Primer CCCTCACACTCAGATCATCTTCT  Reverse Primer GCTACGACGTGGGCTACAG  *Cox-2* Forward Primer TGAGCAACTATTCCAAACCAGC  Reverse Primer GCACGTAGTCTTCGATCACTATC  *iNos* Forward Primer GTTCTCAGCCCAACAATACAAGA  Reverse Primer GTGGACGGGTCGATGTCAC  *Icam-1* Forward Primer GTGATGCTCAGGTATCCATCCA  Reverse Primer CACAGTTCTCAAAGCACAGCG  *Tac1* Forward Primer TGGTGACCTCCCCAGAAGTA  Reverse Primer GTGAGAGAGACGCACAGGAG  *Cgrp* Forward Primer GCTCACCAGGAAGGCATCA  Reverse Primer CATGCCTGGTACAGGAGCAA  *Ccl2* Forward Primer CCAATGAGTAGGCTGGAGAGC  Reverse Primer TCTCATTTGGTTCCGATCCAGG  *Ccl21* Forward Primer GTGATGGAGGGGGTCAGGA  Reverse Primer GGGATGGGACAGCCTAAACT  *Hmgb1* Forward Primer CGGAGAAACTTCAGACCGGA  Reverse Primer CCCATGTTTAGTTGATTTTCCAGC  *Cx3cl1* Forward Primer CTACTAGGAGCTGCGACACG  Reverse Primer AAGCCACTGGGATTCGTGAG  *Tlr2* Forward Primer TCCTCTTCAGCAAACGCTGT  Reverse Primer TTCATGGCTGCTGTGAGTCC  *Tlr4* Forward Primer AATCCCTGCATAGAGGTGTGA  Reverse Primer TCTCCACAGCCACCAGATTC  *Ager* Forward Primer GAAGAGGGGCAGACAGAACC  Reverse Primer ACCAGCTACAGCTCCCCATA  *Actin*  Forward Primer GACGGCCAGGTCATCACTATTG  Reverse Primer CCACAGGATTCCATACCCAAGA |

**Table S2. List of antibodies used in this study.**

| Target antibody | Manufacturer | Species  source | Catalog no. | Application/dilution |
| --- | --- | --- | --- | --- |
| CD68  IBA1  IL-6  TNF-α  NeuN  NeuN  MAP2  Caspase-3  HMGB1  Beta Tubulin  Beta Actin  P65-NF-κB | BIO-RAD  Wako  CST  CST  Merck Millipore  Merck Millipore  Proteintech  CST  CST  Proteintech  Proteintech  CST | Rat  Rabbit  Rabbit  Rabbit  Mouse  Rabbit  Rabbit  Rabbit  Rabbit  Mouse  Mouse  Rabbit | MCA1957  019-19741  12912S  11948S  MAB377  ABN78  17490-1-AP  9662S  3935S  66240-1-Ig  60008-1-Ig  8242S | IHC/1:500  ICC/1:500  IHC/1:1000  ICC/1:1000  ICC/1:500  ICC/1:500  IHC/1:1000  IHC/1:1000  ICC/1:1000  IHC/1:500  WB/1:1000  IHC/1:500  ICC/1:500  ICC/1:2000  WB/1:5000  ICC/1:500 |

WB, Western blot; ICC, Immunocytochemistry; IHC, Immunohistochemistry.

**Table S3. List of gRNA sequences used for CRISPR/Cas9-mediated gene knockout.**

| Target | Number Sequence |
| --- | --- |
| HMGB1  HMGB1  HMGB1  HMGB1  HMGB1  TLR2  TLR2  TLR2  TLR4  TLR4  TLR4  RAGE  RAGE  RAGE | 1 GGAGATCCTAAAAAGCCGAGAGG  2 GAAGTGCTCAGAGAGGTGGAAGG  3 TCATAAGCTCATACTCACGGAGG  4 AGCTCATAAGCTCATACTCACGG  5 GGAGATCCTAAAAAGCCGAGAGG  1 TCACTACGTCTGACTCCGAGGGG  2 GTGCATTCCTCAGACGCTGGAGG  3 AAATGCTGGGAGAACGAGCAGGG  1 ATTCTCCCAAGATCAACCGATGG  2 TCTGACGAACCTAGTACATGTGG  3 TCAGTATCAAGTTTGAGAGGTGG  1 CCACTGGAATTGTCGATGAGGGG  2 CCCATCCAACTCCGAGTCAGGGG  3 GTGGAATAGTCGCTCCTGGTGGG |

**Table S4. List of oligonucleotide sequences used for plasmid construction.**

| Target | Number primers Sequence |
| --- | --- |
| HMGB1  HMGB1  HMGB1  HMGB1  HMGB1  TLR2  TLR2  TLR2  TLR4  TLR4  TLR4  RAGE  RAGE  RAGE | 1 Forward Primer CACCGGGAGATCCTAAAAAGCCGAG  Reverse Primer AAACCTCGGCTTTTTAGGATCTCCC  2 Forward Primer CACCGGAAGTGCTCAGAGAGGTGGA  Reverse Primer AAACTCCACCTCTCTGAGCACTTCC  3 Forward Primer CACCGTCATAAGCTCATACTCACGG  Reverse Primer AAACCCGTGAGTATGAGCTTATGAC  4 Forward Primer CACCGAGCTCATAAGCTCATACTCA  Reverse Primer AAACTGAGTATGAGCTTATGAGCTC  5 Forward Primer CACCGGGAGATCCTAAAAAGCCGAG  Reverse Primer AAACCTCGGCTTTTTAGGATCTCCC  1 Forward Primer CACCGTCACTACGTCTGACTCCGAG  Reverse Primer AAACCTCGGAGTCAGACGTAGTGAC  2 Forward Primer CACCGGTGCATTCCTCAGACGCTGG  Reverse Primer AAACCCAGCGTCTGAGGAATGCACC  3 Forward Primer CACCGAAATGCTGGGAGAACGAGCA  Reverse Primer AAACTGCTCGTTCTCCCAGCATTTC  1 Forward Primer CACCGATTCTCCCAAGATCAACCGA  Reverse Primer AAACTCGGTTGATCTTGGGAGAATC  2 Forward Primer CACCGTCTGACGAACCTAGTACATG  Reverse Primer AAACCATGTACTAGGTTCGTCAGAC  3 Forward Primer CACCGTCAGTATCAAGTTTGAGAGG  Reverse Primer AAACCCTCTCAAACTTGATACTGAC  1 Forward Primer CACCGCCACTGGAATTGTCGATGAG  Reverse Primer AAACCTCATCGACAATTCCAGTGGC  2 Forward Primer CACCGCCCATCCAACTCCGAGTCAG  Reverse Primer AAACCTGACTCGGAGTTGGATGGGC  3 Forward Primer CACCGGTGGAATAGTCGCTCCTGGT  Reverse Primer AAACACCAGGAGCGACTATTCCACC |

**Table S5. List of primers used for the verification of CRISPR/Cas9-mediated gene knockout.**

| Target | Number primers Sequence |
| --- | --- |
| TLR2  TLR2  TLR2  TLR4  TLR4  TLR4  RAGE  RAGE  RAGE | 1 Forward Primer CTCGGAGTCAGACGTAGTGA  Reverse Primer AATAGAACTGGGGGATATGCAAC  2 Forward Primer GTGCATTCCTCAGACGCTGG  Reverse Primer CAGCAACACAGGGAACAACG  3 Forward Primer TGCTCGTTCTCCCAGCATTT  Reverse Primer AGAGAGGTCAGGTTTTTCAGAG  1 Forward Primer TCGGTTGATCTTGGGAGAAT  Reverse Primer TTCGCCAAGCAATGGAACTT  2 Forward Primer TCTGACGAACCTAGTACATG  Reverse Primer AGAGTCAGTTCATGGAGCTT  3 Forward Primer CATGGCTTACACCACCTCTC  Reverse Primer TTTGTCTCCACAGCCACCAG  1 Forward Primer CCACTGGAATTGTCGATGAG  Reverse Primer CGGACTCGGTAGTTGGACTT  2 Forward Primer CCCATCCAACTCCGAGTCAG  Reverse Primer CCACCAGGAGCGACTATTCC  3 Forward Primer GTGGAATAGTCGCTCCTGGT  Reverse Primer AGGGTGCACCATCCTTTATCC |
